# Nucleotide detection mechanism and comparison based on low-dimensional materials: A review

**DOI:** 10.3389/fbioe.2023.1117871

**Published:** 2023-03-02

**Authors:** M. Mustafa Azeem, Muhammad Shafa, Muhammad Aamir, Muhammad Zubair, Basma Souayeh, Mir Waqas Alam

**Affiliations:** ^1^ Department of Civil, Architectural, and Environmental Engineering, Missouri University of Science and Technology, Rolla, MO, United States; ^2^ Yunnan Key Laboratory of Metal-Organic Molecular Materials and Devices, Kunming University, Kunming, Yunnan, China; ^3^ Department of Basic Science, Deanship of Preparatory Year, King Faisal University, Hofuf, Saudi Arabia; ^4^ Mechanical and Nuclear Engineering Department, University of Sharjah, Sharjah, United Arab Emirates; ^5^ Department of Physics, College of Science, King Faisal University, Al Ahsa, Saudi Arabia

**Keywords:** detection, deoxyribonucleic acid, sensors, polymerase chain reaction, surface plasmon resonance, field effect transistor, nucleotide sensors

## Abstract

The recent pandemic has led to the fabrication of new nucleic acid sensors that can detect infinitesimal limits immediately and effectively. Therefore, various techniques have been demonstrated using low-dimensional materials that exhibit ultrahigh detection and accuracy. Numerous detection approaches have been reported, and new methods for impulse sensing are being explored. All ongoing research converges at one unique point, that is, an impetus: the enhanced limit of detection of sensors. There are several reviews on the detection of viruses and other proteins related to disease control point of care; however, to the best of our knowledge, none summarizes the various nucleotide sensors and describes their limits of detection and mechanisms. To understand the far-reaching impact of this discipline, we briefly discussed conventional and nanomaterial-based sensors, and then proposed the feature prospects of these devices. Two types of sensing mechanisms were further divided into their sub-branches: polymerase chain reaction and photospectrometric-based sensors. The nanomaterial-based sensor was further subdivided into optical and electrical sensors. The optical sensors included fluorescence (FL), surface plasmon resonance (SPR), colorimetric, and surface-enhanced Raman scattering (SERS), while electrical sensors included electrochemical luminescence (ECL), microfluidic chip, and field-effect transistor (FET). A synopsis of sensing materials, mechanisms, detection limits, and ranges has been provided. The sensing mechanism and materials used were discussed for each category in terms of length, collectively forming a fusing platform to highlight the ultrahigh detection technique of nucleotide sensors. We discussed potential trends in improving the fabrication of nucleotide nanosensors based on low-dimensional materials. In this area, particular aspects, including sensitivity, detection mechanism, stability, and challenges, were addressed. The optimization of the sensing performance and selection of the best sensor were concluded. Recent trends in the atomic-scale simulation of the development of Deoxyribonucleic acid (DNA) sensors using 2D materials were highlighted. A critical overview of the challenges and opportunities of deoxyribonucleic acid sensors was explored, and progress made in deoxyribonucleic acid detection over the past decade with a family of deoxyribonucleic acid sensors was described. Areas in which further research is needed were included in the future scope.

## 1 Introduction

Deoxyribonucleic acid (DNA) carries the genetic information constituent of deoxyribose and nitrogenous bases known as nucleotides or fragments of DNA (Wilkins, 1956). These nucleotides contain genetic information that can encode life (Stegmann, 2005). As its size is comparable to the nanoscale, the double helix structure was reported in 1953 by Watson and Crick (1953). This well-known DNA double helix is formed from pairs of complementary single-stranded DNA (ssDNA). Double-stranded DNA (dsDNA) is a pair of bonded ssDNA (Watson and Crick, 1953).

Structural nanotechnology has made significant progress in terms of the rapid sensitivity of DNA sequences (Zhou et al., 2020). Each existing DNA is unique, indicating that DNA sequence is crucial for detecting, and it has been studied in the fight against sporadic pandemics. A small part of the DNA that holds genetic information is known as a gene. A complete DNA sequence of DNA is imperative for the fabrication of vaccines. Over the past two decades, many new methodologies have been developed for the detection of DNA, which has ultimately assisted in portable point-of-care diagnostics. Indigenous DNA is negatively charged (Zhang et al., 2007), which can be manipulated by several strategies, such as creating an electric field; thus, it behaves like electrons to attract positively charged particles (Liu and Hu, 2007). Therefore, the identification of specific DNA sequences is a crucial task achieved using modern nanotechnology, which consequently opens a new era of exploration ranging from detection to gene mutation. DNA sensors are capable of detecting changes in the form of electrical signals generated through the immune system in response to any perturbation. They convert biochemical reactions into a signal for further detection. Currently, integrated multiscale simulation and experimental techniques are used to study biosensing applications in a variety of interdisciplinary fields (Quan et al., 2018; Mohammadi et al., 2021; Babar et al., 2022).

Low-dimensional (LD) materials are a class of materials with extraordinary characteristics (Babar et al., 2022). They include graphene and carbon nanotubes (CNT), MXenes (Ti_2_C_3_), hexagonal boron nitride (h-BN), molybdenum disulfide (MoS_2_), and reduced graphene oxide (rGO), *etc.* (Mohammadi et al., 2021). They have displayed they are being used in a variety of applications. Currently, LD based sensors are replacing traditional sensors (Wu et al., 2022). LD materials are used for protective coating and biosensors due to their tunable, electrical, and optical, and excellent mechanical properties. They are also being used as substrate materials in electronic sensors due to their multilayered structures. Additionally, these materials modify their surface chemistry through associated functional groups due to which they respond to a specific analyte (Bolotsky et al., 2019). Other than normal 2D structures, very new types of nanostructured materials with improved optical properties have been reported (Kim and Benelmekki, 2019). They have diverse industrial applications, particularly suitable for biomolecular sensing. These are known as “smart nanosheets or nanoscrolls” (Kim et al., 2014; Kim and Benelmekki, 2018; Kim and Benelmekki, 2019; Benelmekki and Kim, 2023).

**FIGURE 1:**
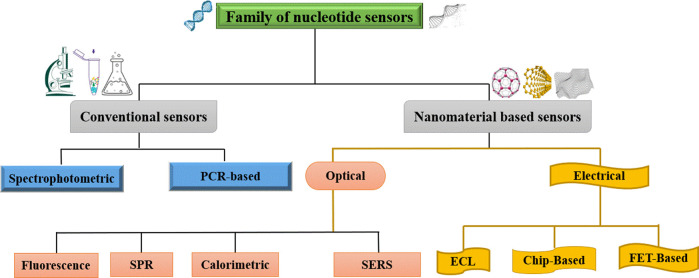
Division of nucleotide sensors.

To achieve global market demand, the fabrication of DNA sensors should possess a low cost, low range of detection (LOD), simple and low processing time with high speed, and high selectivity. There are two main types of DNA detection techniques: conventional spectrophotometric and polymerase chain reactions (PCRs), and modern nanomaterial-based sensors, which are further subdivided into optical and electrical sensors. Optical sensors based on fluorescence (FL), surface plasmon resonance (SPR), colorimetric, and surface-enhanced Raman scattering (SERS) techniques, whereas electrical sensors based on electrochemical luminescence (ECL), chip-based, and field-effect transistors (FET) are described in Figure 1.

Detection methods based on electrical sensors have been gaining attention in the global market owing to their high multiplexing capability, high sensitivity, and wide dynamic range. The major contribution of biosensor involves a transduction mechanism for detection. The transducer transforms the electrical signal from the analyte and amplify it. Conventional sensors have low accuracy and expensive and complex instrumentation whereas nanomaterial-based sensors are more compatible and provide a proficient detection. However, the optical method is commonly used because it has numerous defects, such as it is difficult to reliably profile low-abundance genes, and it requires expensive fabrication of these optical devices and complex bioinformatics tools for fluorescence signal identification. FET-based nano electronic devices have attracted much attention owing to their small scale, simple design, and better performance compared with conventional devices.

**FIGURE 2:**
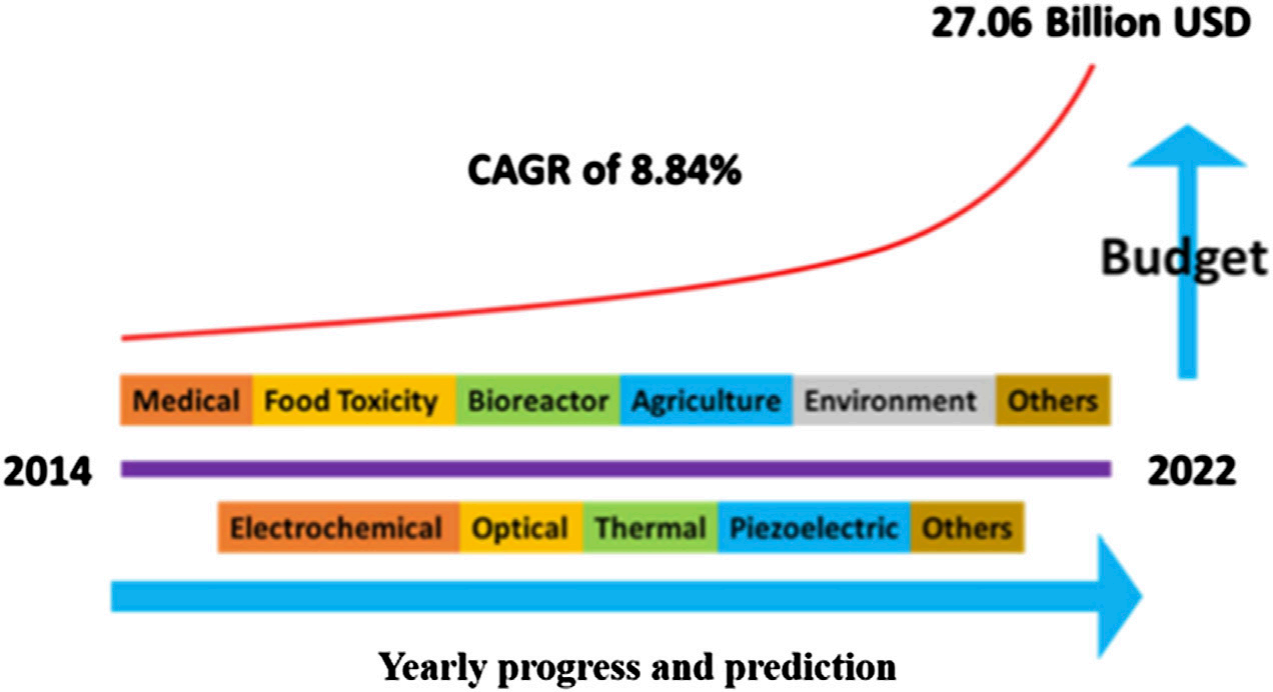
Representation of biosensors in the US industry and annual budget expenses (Mujawar et al., 2020).

Because of the discovery of DNA sensors, efforts have been made to demonstrate and optimize them for portable point-of-care applications. The continuous demand and advancement of nanotechnology has been pointed out by experts, and the DNA sensor market is predicted to reach up to 28 billion dollars. This set an annual growth rate of 8.4% in 2022, as illustrated in Figure 2. In particular, Figure 2 represents data rooted from Scopus using search title as “DNA/RNA/Nucleic acid sensor” and immunosensor/antibody sensor, and “Enzyme’’ sensor (Mujawar et al., 2020).

## 2 DNA sensors and types

A sensor is a combination of receptor-transducer, which converts the biochemical response to signals emitted from the source. Moreover, DNA sensors monitor involving biomolecular processes. A biosensor consists of an analyte, bioreceptor, transducer, electronics, and display setup (Naresh and Lee, 2021).

### 2.1 Conventional sensors (Cs)

#### 2.1.1 Spectrophotometric sensor (Sps)

The interaction of light with matter, known as spectrometry, is an ancient technique that has been used in the fields of chemistry and biomedicine because of its low cost, simplicity, and convenience. This has led to the analytical detection of DNA derivatives, such as nucleic acids and nucleotides, owing to the presence of double bond systems as a consequence of these bonds responding to ultraviolet light in the spectrum. This type of specific absorbance will be helpful for the quantitative measurement of DNA by spectrophotometry in the form of micrograms/mL. The absorbance phenomenon corresponds to the transition of electrons from either the anti-bonding (π*) state to the non-bonding (n) state or from the anti-bonding (π*) state to the bonding state (π) associated with a specific energy (Mujawar et al., 2020). Infect nucleic acids have maximum absorbance of UV light at a wavelength of 260 nm; thus, a solution of DNA is exposed to quantify the exposed sample. The drawback of this methodology is that there is a considerably weak signal for single-stranded nucleic acids owing to the lack of bonding, anti-bonding, and non-bonding states. Furthermore, this technique cannot describe the detailed sequences of nucleic acids. As nucleic acids absorb UV light at 260 nm, the compound containing them absorbs UV at 280 nm. The purity of the sample can be calculated by the ratio of absorbance measurement following the criteria for being pure, i.e., $\frac{A_{260}}{A_{280}}>1.7$ (Shen, 2019).

#### 2.1.2 Polymerase chain reaction-based sensor (PCRs)

Polymerase chain reaction (PCR) is an *in vitro* technique that can detect and amplify small DNA segments. Through this technique, millions of copies are developed in a fraction of the time. This method was proposed in early 1980 (Cantor, 1998), and has been used globally in various fields, such as food quality insurance, genetics, molecular biology, and forensic science. It is a well-established amplification technique during which millions of amplitudes are generated from a small segment of DNA in a short interval of time. Although it is a low-cost and universally used method, constraints, such as indirect melting temperature measurements and premier annealing, hinder their applications. Furthermore, specific amplification will only occur within a narrow range of reaction conditions that ultimately cause the failure of the PCR testing methodology.

**FIGURE 3:**
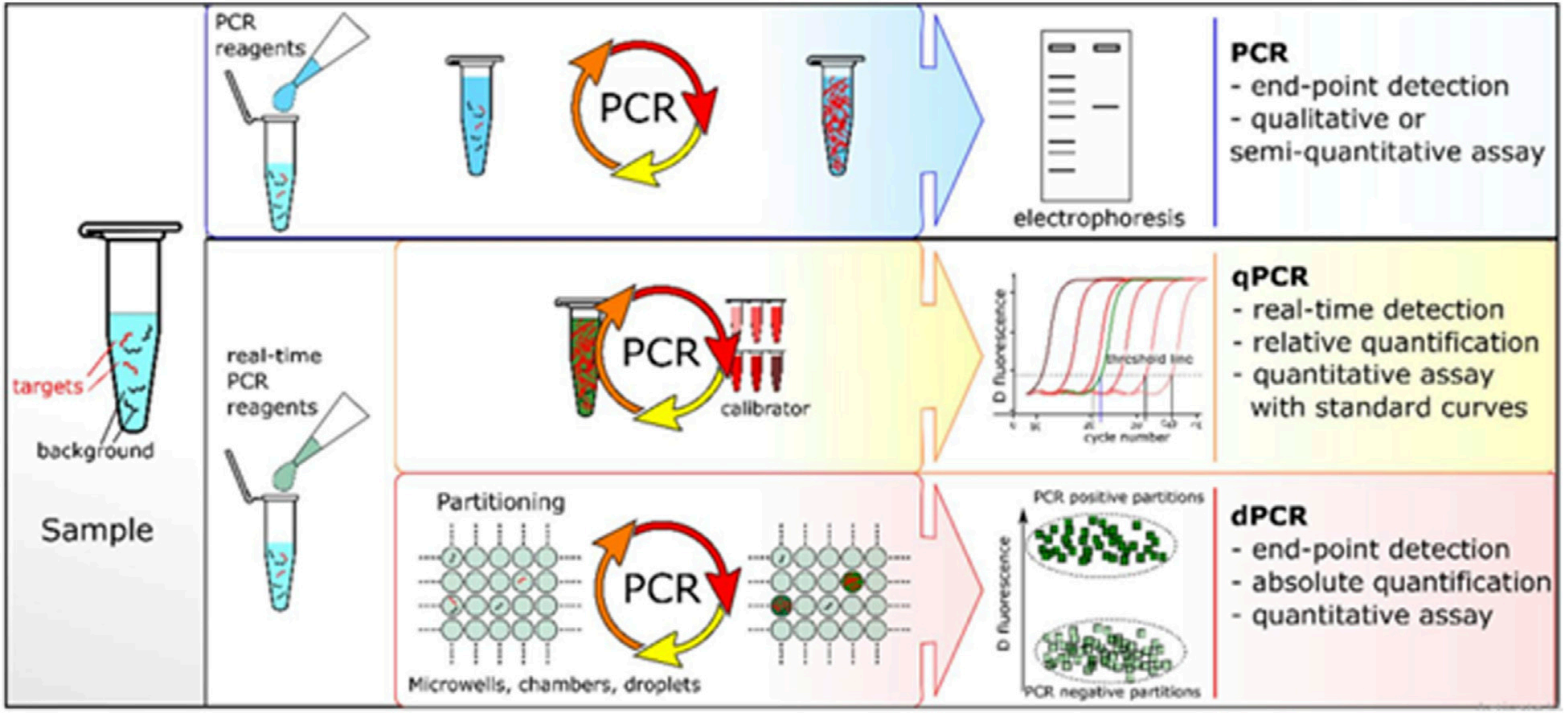
Comparison of different PCR-based techniques (Quan et al., 2018).

For the sample preparation, errors in the volume of reagents significantly alter the result of the polymerase chain reactions cycle that may disrupt the melting temperature, premier annealing, and amplification. All of the building blocks of PCR hinder portability, as these types of measurements cannot be performed outside the laboratory in a well-controlled environment. Signal transduction mechanisms are another significant barrier that prevents PCR from becoming a popular portable device without the use of expensive instruments, such as PCR machines, and complicated processes, including electrophoresis. All these challenges limit the use of PCR as a portable device. Therefore, a simple modification was introduced using a colorimetric assay for the reserved transcription of DNA fragments known as real-time PCR (rt-PCR). RT-PCR was first described in 1990, and a schematic description is shown in Figure 3, which displays a comparison of the different PCR techniques. Conventional PCR, also known as end-point PCR for analysis of DNA amplification, was conducted at the end of fluorescent marking, whereas amplified DNA was analyzed during each cycle, also known as real-time PCR (Wang et al., 2018b). In general PCR, the fragmentation of DNA is pulled through the gel matrix using a centrifugal electric field that separates DNA segments. This process is called electrophoresis. The second PCR test known as qPCR is quantitative PCR, and it is a much more dynamic range of analysis than that of conventional PCR. It is also a modified form of PCR to qualitatively analyze the DNA by the introduction of florescent dyes during PCR cycles. The output of rPCR is typically displayed in the form of sinusoidal followed by a plateau. Digital PCR (dPCR) follows the random distribution of particles over numerous partitions. Each partition acts as an individual PCR through fluorescence detection. Poisson’s statistics were applied to the sample partitions to calculate the concentration of the target sequence from the proportion of the amplified positive concentration. The process constitutes independent partitioning and amplification followed by florescence detection.

### 2.2 Nanomaterial’s base sensors (NMBs)

Nanomaterial-based sensors are considered efficient sensors with excellent signal absorption strength due to quantum effects. They have a high surface-to-volume ratio and higher optical and magnetic properties which make them reasonably good for sensing all types of analytes (Santhanam et al., 2020). Numerous NMBs have been used for DNA biocompatibility because of their higher signal detection capability and transduction technology which converts signals from an analyte during its biochemical reaction (Hlongwane et al., 2019). DNA sensing using NMBs has been included in the following sections.

#### 2.2.1 Optical sensors (Ops)

These biosensors measure changes in optical properties, such as resonance, reflectance, absorbance, and luminescence from the sensor surface. This system measures the fluorescence from nanomaterials based on the detection of DNA hybridization (Vikrant et al., 2019). The optical sensors have been attracted due to their efficient detection level. The materials used in these detectors effectively amplify the detection signal. We summarized a few optical techniques used for developing nanomaterial-based DNA biosensors.

##### 2.2.1.1 Fluorescence-based DNA sensors (FBs)

**FIGURE 4:**
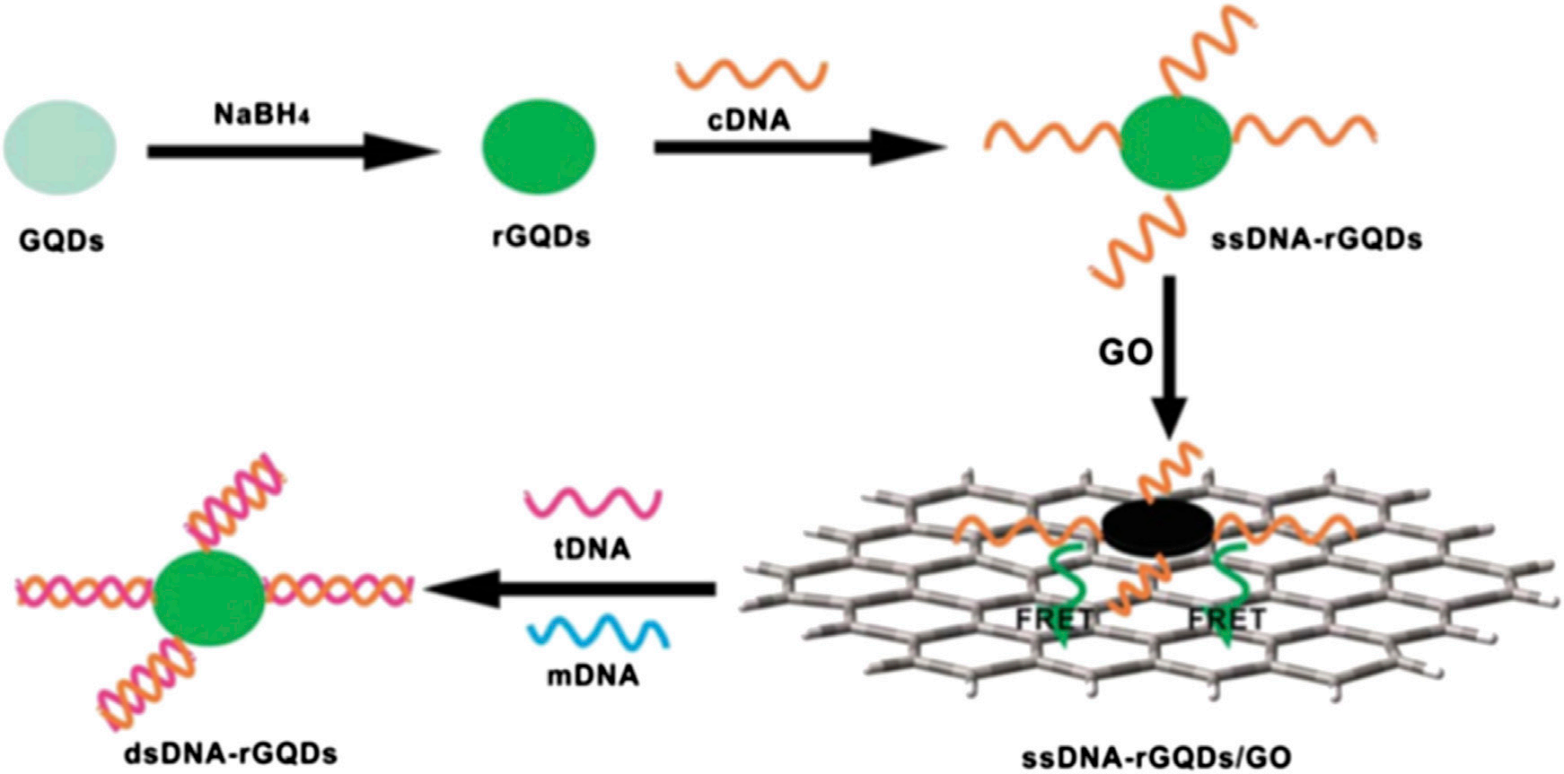
Schematic of a universal fiuorescence based sensor mechanism for the detection of DNA -FRET between GQDs and graphene oxide (GO) (Qian et al., 2014).

In such sensors, a fluorescent nanomaterial, known as the transducer, is conjugated with a DNA molecule acting as a target molecule. A fluorescence spectrophotometer was used to measure the fluorescence emitted from the nanomaterial conjugated with the target DNA, which is known as fluorescence resonance energy transfer (FRET) (Ploetz et al., 2016). In this technique, excited electrons were transferred from DNA to nanomaterials at resonance separated by a nanoscale distance without emission of a photon; this process is called fluorescence quenching (FQ). In the presence of the probe and target DNA, both combine to form a hybrid that leads to a change in the fluorescence intensity of the nanomaterials, similar to the aptamer/DNA sensor. Figure 4 shows a scheme of target detection based on FRET with reduced graphene quantum dots (GQDs) combined with probe (complementary) DNA (single-stranded). A spectrophotometer was used to measure the resonance intensity at the graphene monolayer surface. The FRET signal emerging from the layer was analyzed for the DNA structures. Initially, inorganic GQDs reaction with NaBH_4_ (sodium borohydride) results in reduced GQDs called (rGQDs) followed by connecting DNA (cDNA). This reaction is further divided into three steps. In the first step single strand DNA (ssDNA) interact with GQDs *via* a condensation reaction. Later, this interaction is absorbed through electrostatic stacking making a base pair of ssDNA-rGQDs/GO. Lastly, the base pair interact with the target DNA (tDNA). This tDNA is replaced by a single-based mismatched DNA (mDNA) making a double-strand DNA (dsDNA)-rGQDs compound detached to produce florescence recovery (Qian et al., 2014).

An overview of the variety of recently reported FRET-based DNA detection techniques is listed in Table 1. This table shows the complete information reading type of materials used, techniques and strategies of DNA detection, limit of detection, and range of analyte concentration. Herein, we can conclude that by using a tetrahedral DNA framework, we can achieve a DNA detection limit of 1 fM (Li et al., 2020b) while using Cu (I)-catalyzed alkyne-azide cycloaddition (CuAAC) as a fluorescent nanomaterial can achieve a detection range of 0.22 fM reported by Zheng et al. (2020). Furthermore, Zhang D et al. (2020) reported a 23.8 aM level detection limit using the composition of magnetic beads (MBs) that contain phosphate- Zr^4+^-carboxylate/Cu(II) Br/EDTA for lung cancer DNA. Another finding of magnetic nanoparticle-based on poly-enzyme nanobeads fluorescence assay show high detection of 1.6 aM, which is the highest ever reported by FRET techniques (Lapitan et al., 2019).

**TABLE 1:** Overview of FRET-based DNA detection techniques.

Type of Affinity Assay	Techniques/Strategy	LOD	Analyte Range DLR	References
MoS_2_	Fluorescence/van der Waals force b/w DNA and MoS_2_ in quenching	0.0039 U/mL	0.0039 U/mL	Zhao et al. (2020)
AuNP-H1 probes	Fluorescence/Dual signal amplification	47.68 fM	50 fM–100 pM	Zhang et al. (2020d)
Copper Nanocluster	Fluorescence/was quenched b/w Copper and aptamer	4.8 ng/L/L	0.01–1,000 ug	Zhao et al. (2020)
TAE/Mg^+2^ buffer	Fluorescence/isothermal signal amplification	9.8 pM	0.01–10 nM	Zhao et al. (2020)
Silica Nanoparticles	Fluorescence/polydopamine modified SiO_2_ as quenchers	1 nM	0–12 nM	Zhao et al. (2020)
δ-FeOOH nanosheet	Fluorescence/DNA and FeOOH as quenchers	10 pM	0–20 nM	Wu et al. (2020a)
Curcumin Encapsulated with F108	hydrogen bonding and dipole interaction between curcumin and F108 increasing FL to 6 times	50 uM	0–100 μM	Bechnak et al. (2020)
DNA Tetrahedron Nanoprobe	FRET/DNA assisted cyclic amplification	6 pM	10 pM–100 nM	Gao et al. (2020a)
Tetrahedral DNA Framework	Fluorescence/developed bridge DNA sensors that can capture target DNA	1 fM	0.01 nM–10 nM	Li et al. (2020b)
Carbon Nanoparticles	Fluorescence/developed DNase I-aided cyclic enzymatic amplification method	3.2 pM	25–100 pM	Li et al. (2020d)
Plasmonic gold nanostars	SERS/Fluorescence/developed a dual-modal aptamer-based biosensor	FL: 0.50 μg/L and SERS: 0.77 μg/L	0.1–50 μg/L	Li et al. (2020d)
Poly- nucleotide kinase	Fluorescence/isothermal signal amplification	0.1–0.005 U/mL	3 uU/mL	Li et al. (2020e)
Cy5/Fluorescein	FRET/developed a ratiometric fluorescence method for Four-way Junction	0.12 nM	1–200 nM	Liu et al. (2020b)
G-triplex micro beacons/thioflavin	Fluorescence/Label-free fluorescence method combined G3-MB with Exo III‒aided DNA recycling amplification	5.6 pg/mL	75–750 nM	Liu et al. (2020c)
GO based modified with Cu(II)	PL/graphene oxide-based modified platform that boost photoluminescence	4.6 × 10–7 M	0–20 μM	Pal et al. (2020)
Thioflavin/ErBr	FRET/ThT-fluorescence-based DNA composition studied	1.1 nM	0–4uM	Pramanik et al. (2020)
Carbon Dots and Au Nanoparticles	Fluorescence/A FQ energy transfer as “spectroscopic rulers” between CDs and AuNPs	1.03 ± 3.54 nM	0.01–200 nM	Saad et al. (2020)
Biotinylated sensing probes/magnetic beads	Fluorescence/DNA methyltransferase catalyzes and produce strong fluorescent signalssignals	0.002 U/mL	0.01–10 U/mL	Liu et al. (2020f)
Au nanoparticles on covalent organic framework nanosheets	Fluorescence/Au NPs/COF NSs used as an efficient quencher	75 pM	0.1–10 nM	Tian et al. (2020)
Fe_3_O_4_ MBs/FL	Fluorescence/activation of a hybridization sensor by a magnetic field	2-fold		Bakshi et al. (2017), Bakshi et al. (2019)
Fe_3_O_4_/SiO_2_/Graphene	FRET/graphene oxide-based FQ sensor. (fluorescent magnetic nanoparticles as donor)	0.12 µM	0–10 µM	Balaji et al. (2019)
multicolor QDs open-ring nanoarrays with silver-Plosmonic	FRET/Fluorescence enhancement and quenching can be switched that provide platform for DNA detection	∼300 fM	100 fM^–1^ μM	Kannegulla et al., 2018a, Kannegulla et al., 2018b
		Plane Silver - 6 nM		
MBs/phosphate-Zr^4+^-carboxylate/Cu(II)Br/EDTA	Fluorescence/approach used electron transfer atom transfer radical polymerization with EDTA as the metal ligand	23.8 aM	0.1 fM–1 nM	Zhang J et al. (2020)
Cu (I)-catalyzed alkyne-azide cycloaddition	Fluorescence/Based on controllability and signal amplification by atom transfer radical polymerization	0.22 fM	1 fM–1 nM	Zheng et al. (2020)
MoS_2_, WS_2_, and GO	Fluorescence/fluorescently labeled DNA oligonucleotides were used and their adsorption capacities and kinetics were studied as a function of ionic strength	WS_2_-3nM, MoS_2_-1.5 nM, GO-2nM	1–50 nM	Lu et al. (2017)
Streptavidin-coated magnetic beads/Liposome/carboxyfluorescein	Fluorescent/carboxyfluorescein-loaded liposomes as signal amplification systems strategy for detection of DNA sequence	1 nM	1 × 10^−10^–6×10^-10^ M	Sforzi et al. (2020)
PDMS chip and a glass substrate Microfluidic	Fluorescence/Microfluidic Exponential Rolling Circle Amplification platform	5–8 (zeptomole) 2 × 10^6^ (exosomes)	50 zmol to 5 fmol	Cao et al. (2019)
Polydimethylsiloxane microfluidic device Microfluidic	fluorescence/Develop An ion concentration polarization based electrokinetic concentration device	1 nM (DNA)		Cheung et al. (2018)
		25 nM (RMNA)		
Microfluidic chip/microbead/Microfluidic	fluorescence/developed a rapidly adaptable platform to assess biomarkers using a microfluidic technology	0.5 ng/μL (chip), 0.1 ng/μL (multiwell plate)	0.2 ng/μL–100 ng/μL	Dinter et al. (2019)
Poly-L-Lysine Microfluidic chip/	fluorescence Proposed PLL substrate on with microfluidic chips for detection of DNA based on three-segment hybridizationhybridization	1 pM/30 min	10^−7^M to 10^−12^ M	Gao et al. (2020b)
LOC for free-flow electrophoretic/	Fluorescence/demonstrate DNA amplification by microfluidic method from dilute specimens	1 PFU/mL	5–500 µL	Hügle et al. (2020)
Fe_3_O_4_@PDA NPs by Ca^2+^	FRET/developed FRET-based biosensor for determination of microRNA-167 using CDs as donor and Fe_3_O_4_@PDA NPs as acceptor	76 pM	0.5–100 nM	Cao et al. (2020)
Iron oxide nanocubes microfluidic	Flow cytometry/Designed a new kind of superparamagnetic nanobeacon for mRNA detection and regulation in living cells	9.6-fold	54 nM	Chen et al. (2019)
Magnetic nanoparticle/poly-enzyme nanobead/FL	Detection target DNA by combining magnetic nanoparticle capture and poly-enzyme nanobead signal amplification	1.6 aM	1 aM–1 pM	Lapitan et al. (2019)

##### 2.2.1.2 Surface plasmon resonance-based DNA sensors (SPRs)

**FIGURE 5:**
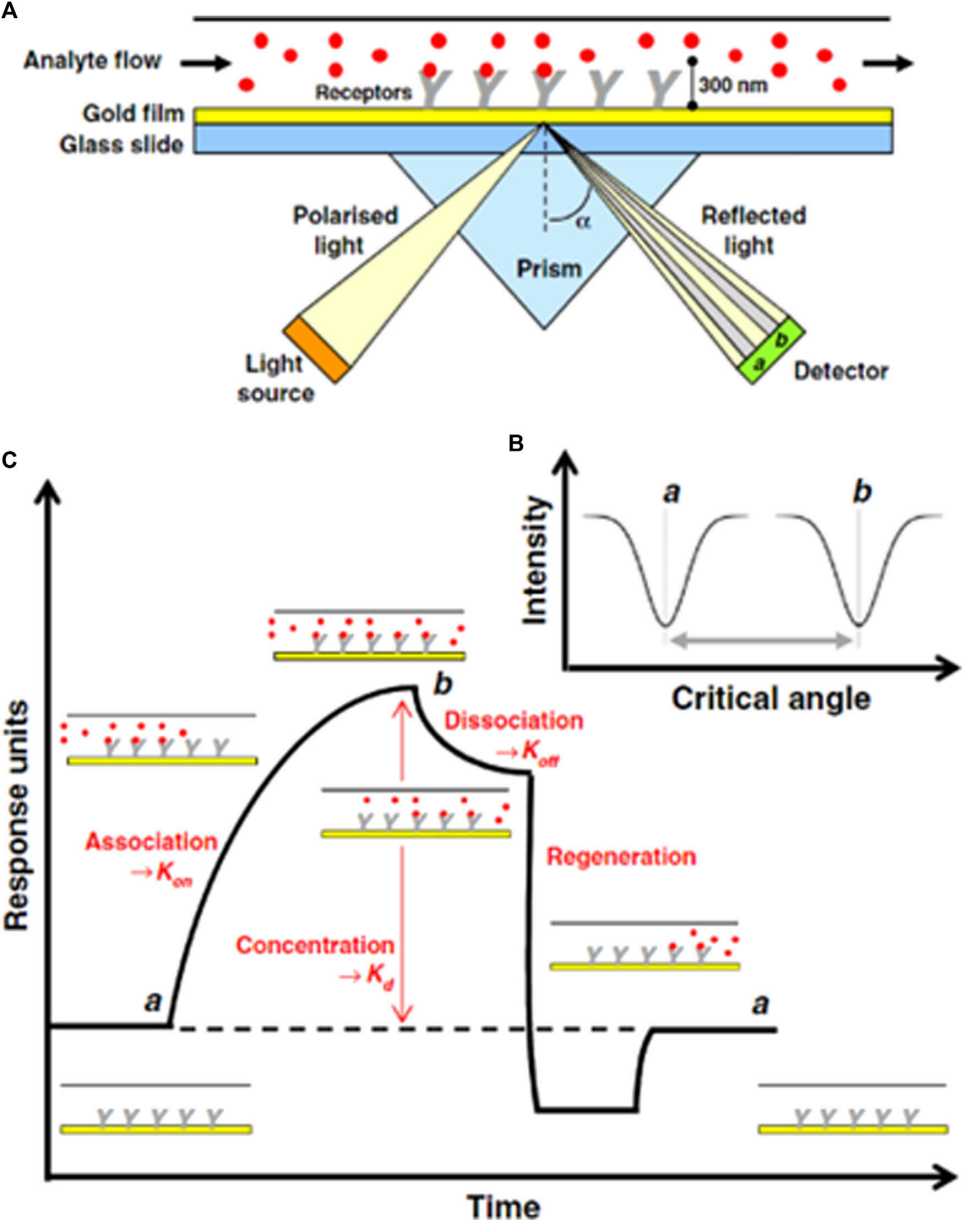
**(A)** Schematic of SPR-based DNA sensing technique for measuring the binding of an analyte molecule **(B)** varation in critical angle as fuction of intensity of incident light, **(C)** Evolution of sonogram's response during SPR experiment (Patching, 2014).

It is one of the most common optical sensing techniques which is based on surface plasmon which are electromagnetic waves originating from the metal interface. In this sensor, the incident light stimulates the resonance of the conduction electrons at the interface of the positive and negative permittivity materials. AuNPs with positive charge conjugate with the target DNA that is negatively charged; light stimulus resonance phenomena occur between them using conduction electrons, and there is a change in dielectric constants that generate surface plasmons. The schematic diagram shows the detection method by SPR, where the prism, transducer surface composed of nanomaterials, plane polarized light as a stimulus, and detector are the main components of the SPR system. During the conversion of the association phase to the disassociation phase, the refractive index changes, which ultimately deviate from the exiting light from the prism, are correlated with the concentration of the analyte, as shown in Figure 5. Specifically, Figure 5 represents the experimental illustration of SPR techniques (A) and a variation in the critical angle as a function of the intensity (B) and the response of the sensor during the experiment. This method offers label-free techniques; however, there is variation in the refractive index owing to changes in the transducer surface temperature and composition, which may significantly alter the detection results (Patching, 2014).

The SPR detection performs the analysis of biomolecular interaction ranging from organic compounds to proteins and nucleic acids and viruses based on real-time. The detection is non-invasive. i.e., it can analyze transparent or colored samples effectively. They are a label-free, specific, and sensitive method that is dependent on the changes in the refractive index of the material surface. A small change in the refractive index may induce a false signal during the detection of an analyte. Nanomaterials are the most ideal material for their fabrication and better signal detection and amplification. Most importantly they are available commercially (Deng et al., 2017; Das et al., 2021).

An overview of the various SPR-based DNA detection techniques reported recently is displayed in Table 2. We have listed all possible information, for example, the type of materials, techniques, and strategy of DNA detection used, limit of detection, and range of analyte concentration. Therefore, by employing a sandwich-like assay based on the selective capture of specific DNA targets, subsequent signal amplification can be obtained by a secondary DNA probe linked to Au nanostars with a detection limit of 3 fM to 6.9 aM (Mariani et al., 2015). Another study reported that a highly sensitive polarization control-modulated plasmonic biosensor based on monolayer graphene with Au film π -staking achieved a detection limit of 500 aM (Sun et al., 2019b), which is the highest ever reported for SPR techniques. Table 2 summarizes the findings based on the SPR-DNA sensor reported previously.

**TABLE 2:** SPRs based DNA detection techniques.

Type of Affinity Assay	Determination Method	LOD	Analyte Range DLR	References
Silica core/gold nanowires	SPR/a novel design of a highly sensitive surface plasmon photonic crystal fiber biosensor for DNA hybridization detection is presented	5.53–6.82 RIU-1	94.59 nm/RIU	Azab et al. (2018)
Au/SiO_2_/H_2_O/L inker	SPR/A highly sensitive hybrid plasmonic slot-waveguide biosensor based on silicon-on-insulator is proposed which record change in the analyte refractive index	2.65 *×* 10^−6^ RIU	1890.4 nm/RIU	Hameed et al. (2017)
Biotinylated thiolated DNA molecular beacon (MB)/streptavidin functionalized gold nanorods	SPR/strategy by applying biotinylated thiolated molecular beacon (MB) interfacial gene probe and a strepavidinylated GNR (Stre-GNR) for the enhanced SPR response to realize rapid and sensitive miRNA detection is demonstrated	0.045 pM	0–2 nM	Hao et al. (2017)
Nanobowled Aluminum/Au NPs	LSPR/fabricated ordered gold nanoparticle arrangements on epoxy substrates is presented	5 nM	1–1,000 nM	Lednický and Bonyár (2020)
Gold nanostar (AuNS)/SPR	SPRi/Designed sandwich-like assay based on the selective capturing of specific DNA targets and the subsequent signal amplification by a secondary DNA probe linked to AuNS	3.0 fM (without NSs) 6.9 aM (with NSs)	6.1 nM–10 pM (without NSs)	Mariani et al. (2015)
			1.5 fM–10 aM (with NSs)	
3D gold nanostructure with Au deposition	LSPR of gold nanoparticles is sensitive to the dielectric constant of the surrounding environment	1–500 nM	13 fM	Na et al. (2018)
Au nanoparticles	LSPR peak is sensitive only to the refractive index of the close surrounding environment	430 nm/RIU	0–100 nM	Qi and Bi (2019)
Graphene/Au NPs	SPR/Sensitive polarization control-modulated plasmonic biosensor based on monolayer graphene at gold film- π -staking interaction	500 aM	10^−15^ to 10^−7^ M	Sun et al. (2019b)
Gold nanorods	Using LSPR/establish a method that can discriminate between the mutant and the wild-type sequence of the gene using gold nanorods in solution	2 ng/mL	0–125 ng/mL	Tadimety et al. (2019)
Inverted-bowtie nanoapertures	SPR/show simultaneous ionic-current and optical-transmission-based detection of DNA	100 nm		Verschueren et al. (2019)
Dual Gold Nanoparticles	SPR/Dual nanoparticle amplification was achieved by controlled hybridization attachment of AuNPs resulting from electronic coupling between the Au film and AuNPs, as well as coupling effects in plasmonic nanostructures	5 × 10^3^ exosomes/mL	10^6^–10^9^ exosomes/mL	Wang et al. (2019b)
Gold NPs	LSPR/AuNPs bind with DNA, this binding changes the local refractive index, which is detected spectroscopically as the resulting changes of the LSPR peak wavelength	60 nM	0–31 nM	Zopf et al. (2019)
Silicon dioxide Microfluidic	LSPR/developed a label-free microfluidic biosensor platform to detect the interaction of DNA with the DNA polymerase enzyme, to monitor the formation of Self-assembled-monolayers of ssDNA	54 ± 6 nm/RIU	0.0625 U/mL	Roether et al. (2019)

##### 2.2.1.3 Colorimetric-based DNA sensors (CMs)

**FIGURE 6:**
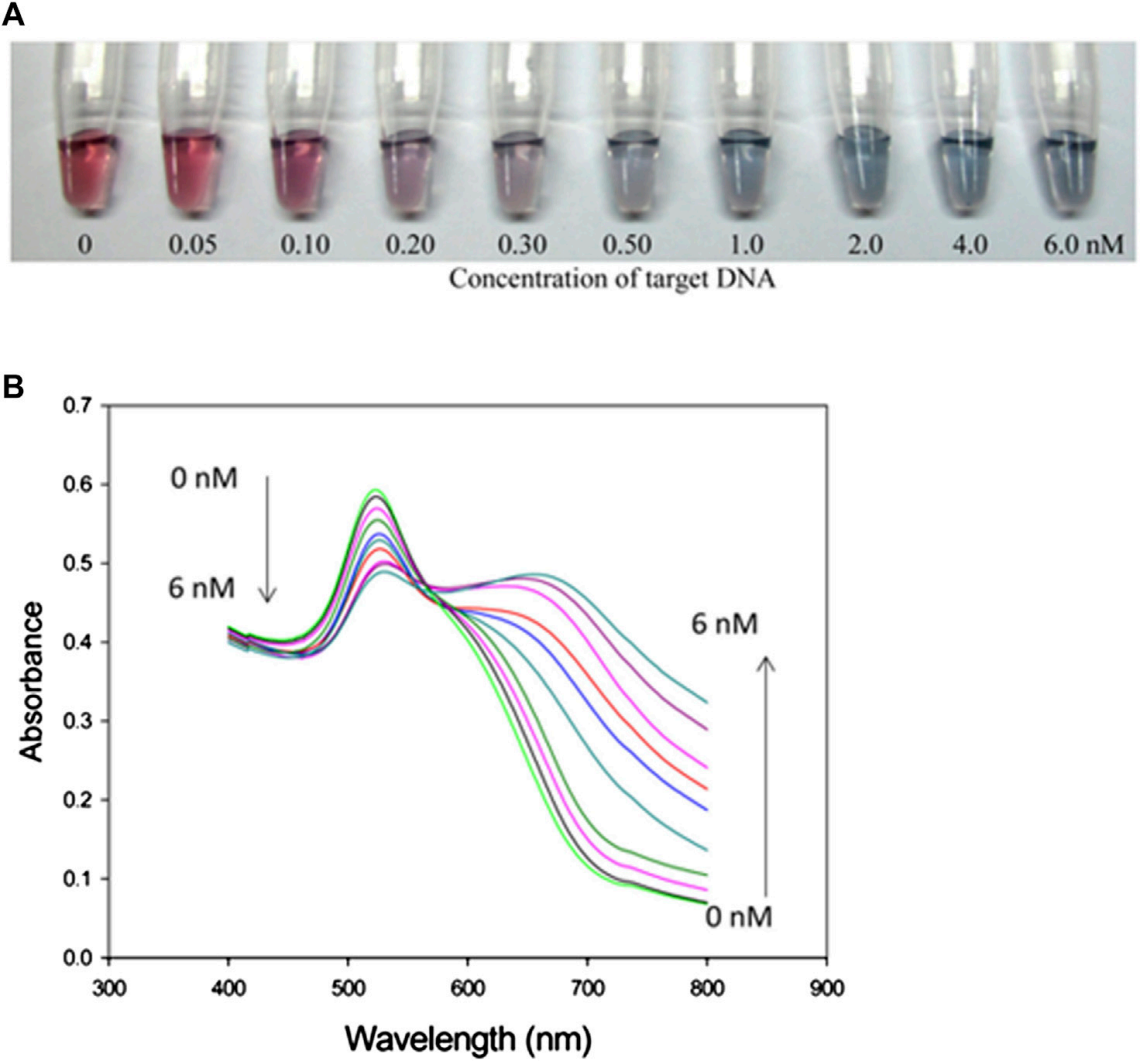
**(A)** Calorimetric response of concentration of DNA (0-6 nM), **(B)** absorption spectrum as a function of wavelength of electrolyzed DNA (Liu et al.,2013).

In this technique, color tags or enzymes are used for the detection of DNA compatible with the substrate. Alternatively, nucleic acids functionalized with nanomaterial-based assays can be used for the detection and quantification of DNA (Krishnan and Syed, 2022). During colorimetric analysis, colloidal solution gold nanoparticles (GNPs) exhibit different colors based on their distance from red to blue. The detection is done through a change in the wavelength of the electrolyzed DNA. The color of GNPs is dependent on their dispersion and this help researchers to visually investigate assays that change owing to a decrease in the average distance between nanomaterials (Li and Rothberg, 2004). This method is straightforward, low-cost, and easy to perform for rapid onsite diagnostics. They have a poor limit of detection and prototype design. This DNA detection equipment is commercially available and more economical than other clinical diagnostics. Figure 6 displays schematic illustration of calorimeter based on the GNPs dispersion and aggregation (Liu et al., 2013). A series of DNA samples were electrolyzed and had different concentrations up to 6Nm. The color of the GNPs is gradually changing from pale to blue with the addition of concentration. The wavelength of absorption spectrum is in the range of 550–750 nm. This method is designed to detect a DNA sequence.

An overview of the various colorimetric DNA detection techniques recently reported is listed in Table 3. We summarized the types of materials used, techniques and strategies of DNA detection, limit of detection, and range of analyte concentration. Therefore, by employing electrophoretic streptavidin-coated MBs assisted with a magnetic field, we could introduce a new method, which uses active hybridization with a detection limit of 0.1 fM (Tian et al., 2019). Another report showed that Fe_3_O_4_ nanosheets in DNA/Fe_3_O_4_ networks display peroxidase-like catalytic activity, thereby enhancing detection to an extreme limit of 13 aM, which is the highest limit that has been reported for colorimetric techniques (Tang et al., 2019).

**TABLE 3:** Colorimetric-based DNA detection techniques.

Type of affinity assay	Determination method	LOD	DLR	References (s)
2D DNA/Fe_3_O_4_ nanosheet	Colorimetry/Fe_3_O_4_ nanosheets in the DNA/Fe_3_O_4_ networks display peroxidase-like catalytic activity thereby enhancing detection	13 aM	0.05 fM–12 nM	Tang et al. (2019)
Fe_2_O_3_ porous particles	Calorimetric/DNA is detected using the peroxidase-like activity of a composite nanozyme synthesized in the form of porous particles	1.1 nM	0.0–21.5 nM	Dinter et al. (2019)
Magnetic microbeads/ nanoparticles	Optomagnetic/isothermal amplification technique	4 and 20 pM	1–100 pM	Minero et al. (2019)
Magnetic microbeads	Optomagnetic/isothermal nucleic acid amplification	4 pM	2–40 pM	Minero et al. (2020)
Iron oxide nanoparticles	Optomagnetic/giant magnetoresistive effect	*p* = 0.05		Ng et al. (2019)
Streptavidin-coated MBs/electrophoresis	Electrophoretic assisted with magnetic field/multiplex microarray-based assay/new method of active hybridization analysis	0.1 fM	0.1 nM	Shlyapnikov et al. (2019)
Magnetic nanoparticle-graphene oxide	Optomagnetic/isothermal nucleic acid amplification triggered by the hybridization and padlock probes	2 pM	1–4 pM	Tian et al. (2019)
Gold nanoparticles probe	Plasma mass spectrometry detection/Au NPs amplification and isothermal circular strand-displacement polymerization reaction	8.9 fM	0.1–10000 pM	Xiao et al. (2019)
Silicon master/Microfluidic	Microfluidic recombinase polymerase amplification (RPA) sensor	10 copies/μL	10–10^6^ copies/μL	Yang et al. (2019)
Peptide Nucleic Acid	PNA-DNA2 triple-helix molecular switch and DiSC_2_	0.18 nM	0–2 μM	Xu et al. (2020a)

##### 2.2.1.4 Surface-enhanced Raman scattering-based DNA sensors (SERSs)

In this technique, molecular vibrations which arise directly from analyte molecules were measured using Raman spectroscopy. Such types of resonance occur only when the target analyte is a nanostructure or a roughed metal surface. SERS sensing is based on the conjugation of nanomaterials and bioreceptor molecules (oligonucleotides) at the surface of a dye known as a Raman reporter or tag that enhances Raman signals during the detection of target DNA. SERS provides an enhanced Raman signal of 10^6^–10^14^ order of magnitude owing to the electromagnetic interaction between the metal.

**FIGURE 7:**
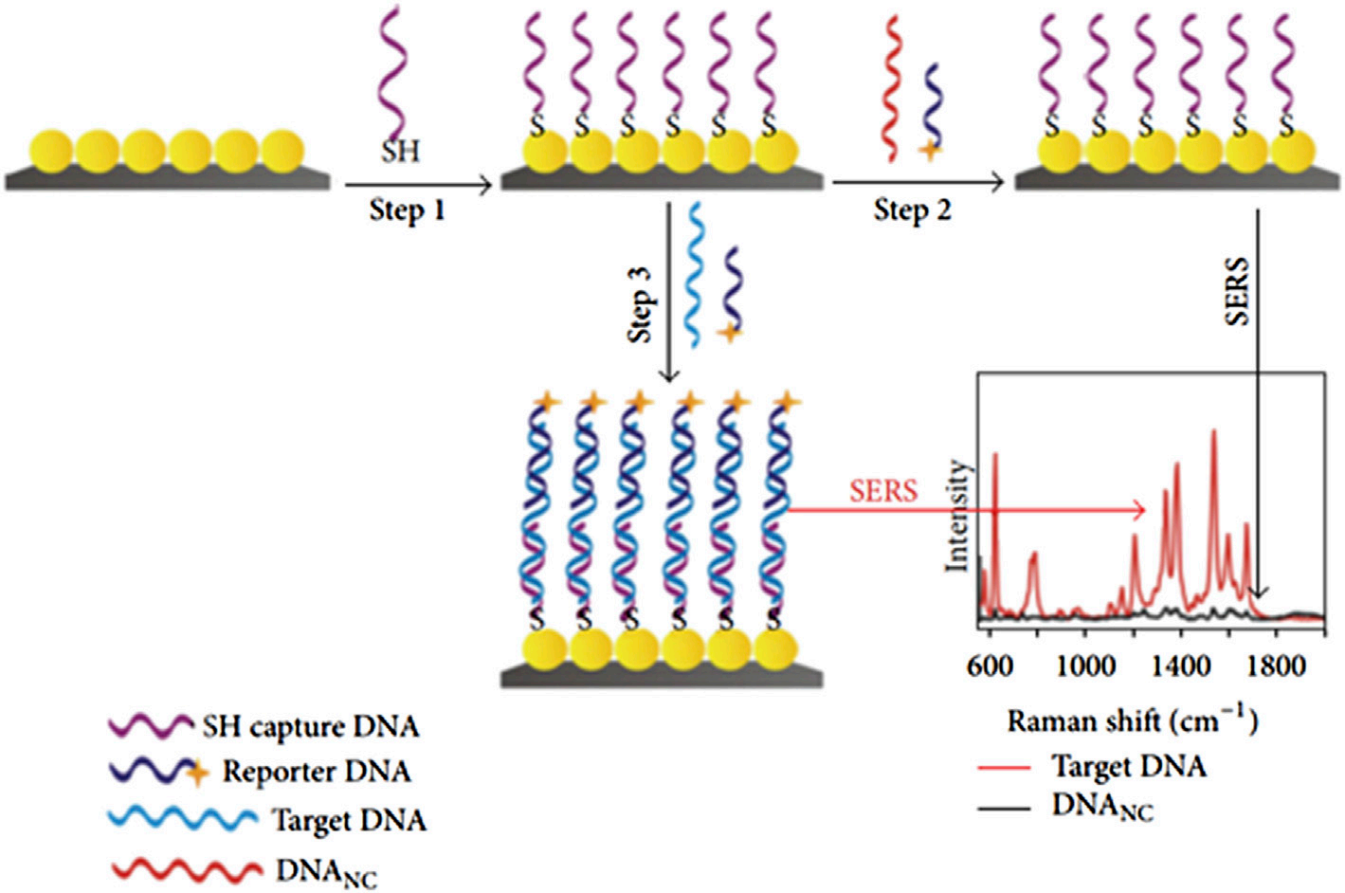
Schematic of the fabrication route for SERS sensor and variation of Raman shift as a function of intensity (Jiang et al., 2012).

**FIGURE 8:**
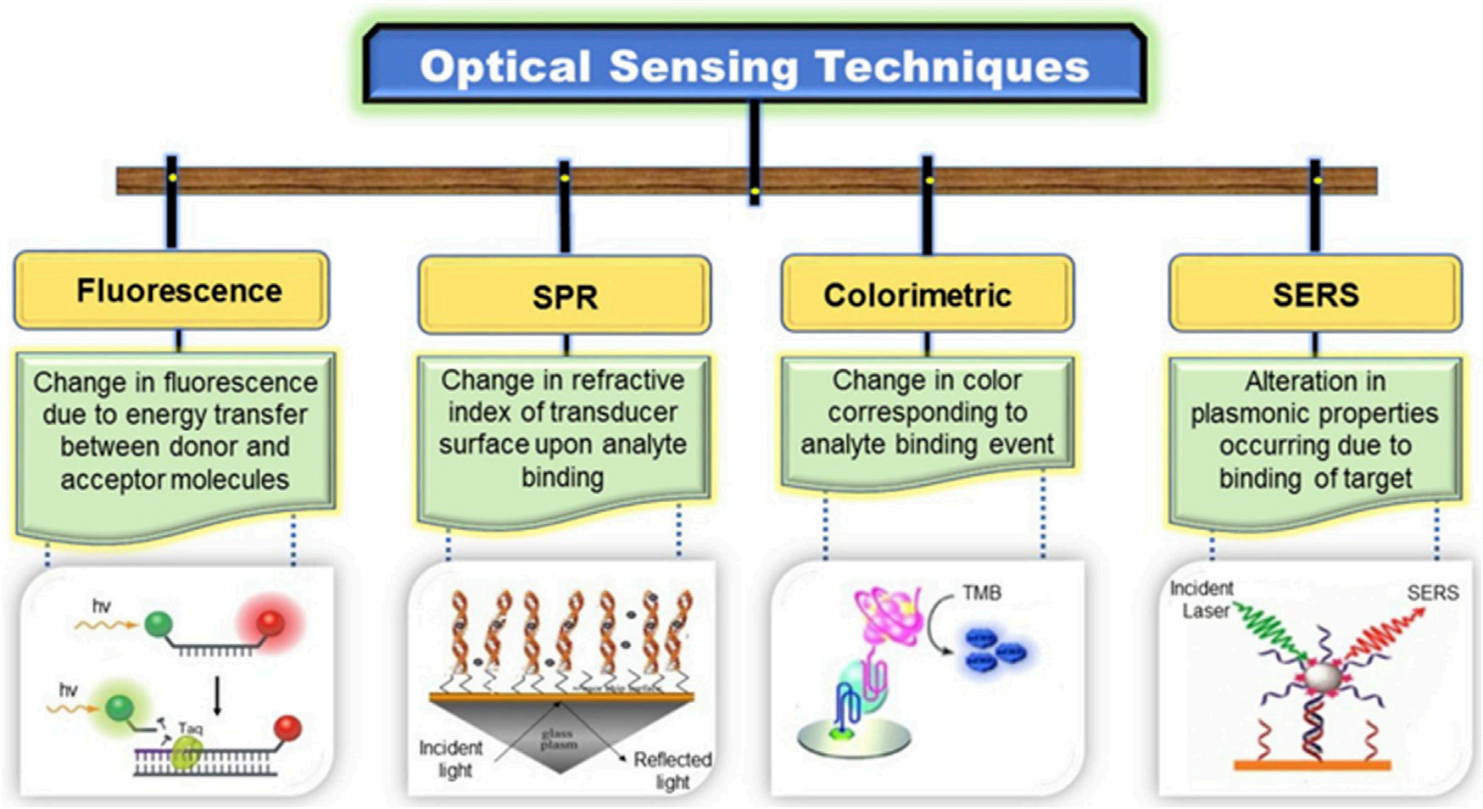
Division of optical sensors (Vikrant et al., 2019).

Nanostructures and the analyte. SERS-based biosensors have a comparatively low cost, high sensitivity, rapid results, and portability (Vikrant et al., 2019). Figure 7 shows the schematic setup for the detection of DNA based on AgNPs at the Si substrate as a sandwich-type sensing setup. Nanoparticles are functionalized with thiolated DNA as step 1, followed by conjugation of dye named Rhodamine as step 2. Therefore, a sandwich-like structure surrounded the target DNA by thiolated DNA and reported DNA that ultimately caused the formation of capture/target/reporter DNA ready for SERS detection as step 3, as shown in Figure 7. The optical sensors are summarized in Figure 8. Each optical sensor has been summarized in the flow chart.

An overview of the various SERS-based DNA detection techniques reported recently is listed in Table 4. It contains the complete information type of materials used, techniques, a strategy of DNA detection, limit of detection, and range of analyte concentration. Thus, a detection limit of 10 uM–10 fM can be achieved by AuNPs on the surface of graphene oxide (GO) linking *via* hybridization (Khalil et al., 2019). He et al. (2019) reported a novel detection ratiometric sensor based on glucose oxidase (GOx) on Au and Si nanoflower substrates that enhances the detection to an extreme limit of 7.75 aM, which is the highest limit for colorimetric techniques. Table 4 lists earlier studies on SERS-based DNA sensors.

**TABLE 4:** SERS-based DNA detection techniques.

Type of affinity assay	Determination method	LOD	DLR	References(s)
DNA Probe-conjugated AuNPs	SERS/Salivary biomarkers S100 calcium binding protein *p* (S100 P) mRNA in saliva is a potential biomarker	3 nM	0–200 nM	Han et al. (2018b)
Au NPs probe	SERS/Colorimetry/developed dual-mode Au NPs probe	1 cell	100–10^6^ cells mL^-1^	Feng et al. (2019)
Ag NPS/SERS	SERS/The simple strategy relies on the electrostatic adhesion of DNA/RNA onto positively charged silver colloids thus providing realistic direct information of the nucleic acid		1 pg μ/L	Guerrini et al. (2016)
Polyadenine/AuNS	SERS/Developed a method for the modulation of DNA conformation from the “Lie-Down” to the “Stand-Up” conformation on a AuNS surface by adjusting the length of tip-assembled polyA grafted to the DNA sequence	45.7 pM	0.1–500 nM	Guo et al. (2017)
Silver nanoparticles and Graphene oxide-based biochips	SERS/Laser scribing method to fabricate biochips as a reusable sensor	10^−5^–0^−10^ M	10^−10^ M	Han et al. (2018a)
Au NPs were conjugated with oligonucleotides	SERS/Developed SERS-based vertical flow assay biosensor	0.01–200 nM	1.1–10 nM	Han et al. (2019)
Glucose oxidase (Gox) on Au & Si nanoflowers substrate	SERS/fabricated ratiometric sensor with only one Raman probe based on cascade catalytic reaction	7.75 aM	10 aM–100 pM	He et al. (2019)
DNA on the AgNPs@Si	SERS/AgNPs@Si based substrates for sensitive,specific,andmultiplexDNA detection	1 pM–100 nm	1 pM	Jiang et al. (2012)
GO-Au NPs/Raman dye	SERS/detection is based on the covalent linking of the two platforms [GO-AuNPs with probe 1 and AuNPs with probe 2 and Raman dye (Cy3) ] *via* hybridization	10 µM–10 fM	10 fM	Khalil et al. (2019)
Metal–organic framework coated Ag-NOF Sensor	SERS/reported that a MOF enables an Ag nanowire SERS platform to be corrosion resistant		1 nM	
Au–Ag bimetallic nanodendrites	Demonstrated SERS-based sensor that utilizes the toehold-mediated DNA displacement reaction as a target-capturing scheme	200 fM–20 nM	96.3 fM	Hyejeong Jang (2019)
GaN/Au Substrate/SERS	SERS/Detection of gene mutation-using highly active and reproducible substrate (photo-etched GaN covered with a thin layer of sputtered gold)	6.75 pg μ/L −67.5 ng μ/L	1 pg μ/L	Kowalczyk et al. (2019)
R6G + AgAu alloy + silicon microbead	SERS/proposed strategy by combining stable SERS reporter element and duplex-specific assisted signal amplification for quantitative detection	12 fM–18 pM	5 fM	Ma et al. (2018)
PDMS chips integrating silver-coated porous silicon membranes	SERS/metal–dielectric nanostructures were functionalized with enzyme-linked immunosorbent assay for the detection of mRNA.	25–1 nM	0.55–1.51 nM	Novara et al. (2017)
Graphene-Ag array	SERS/developed graphene-Ag array for the detection of methylated DNA and its oxidation derivatives	200 pg genomic DNA	1.8 pmol/L	Ouyang et al. (2017)
DNA hydrogel/SERS	SERS developed a novel sensor array with nine sensor units that can detect multiple miRNAs in one sample based on a target miRNA-responsive DNA hydrogel	4–1,200 nM	0.11 nM	Si et al. (2020)
Silver-coated gold nanostar	SERS/*in vivo* detection of nucleic acid involving the “inverse molecular sentinel” detection scheme using plasmonics-active nanostars		5 nM	Wang et al. (2018a)
Co/Au NPs	SERS/PCR/based new platform was proposed and evaluated its performance by sequentially measuring the Raman signals of DNA after the completion of different thermocycling numbers	0.1–1,000 pM	960 nM	Wu et al. (2020b)
Melamine resin/Ag/SiO2 nanoparticles	SERS/FL/dual-mode spectroscopic encoded microspheres system based on the combination of FL and SERS spectra encoding was designed for specific DNA detection		100 uL	You et al. (2017)
			10–10 mol/L	
Silica-coated/AuNPs, and Au-coated NPs with DNA probes	SERS/developed a magnetic-capture-based SERS assay for the simultaneous detection of multiple nucleic basis	120 fM	120–450 pM	Zhang et al. (2019a)
Tetracationic Bis-triarylborane	SER/designed new derivatives to investigate the influence of the linker type on DNA/RNA/protein interactions by fluorimetric titration	10 nM	0.5 nM–0.005 nM	Amini et al. (2020)

#### 2.2.2 Electrochemical sensors (ECs)

##### 2.2.2.1 Electro-chemiluminescence based DNA sensors (ECLs)

**FIGURE 9:**
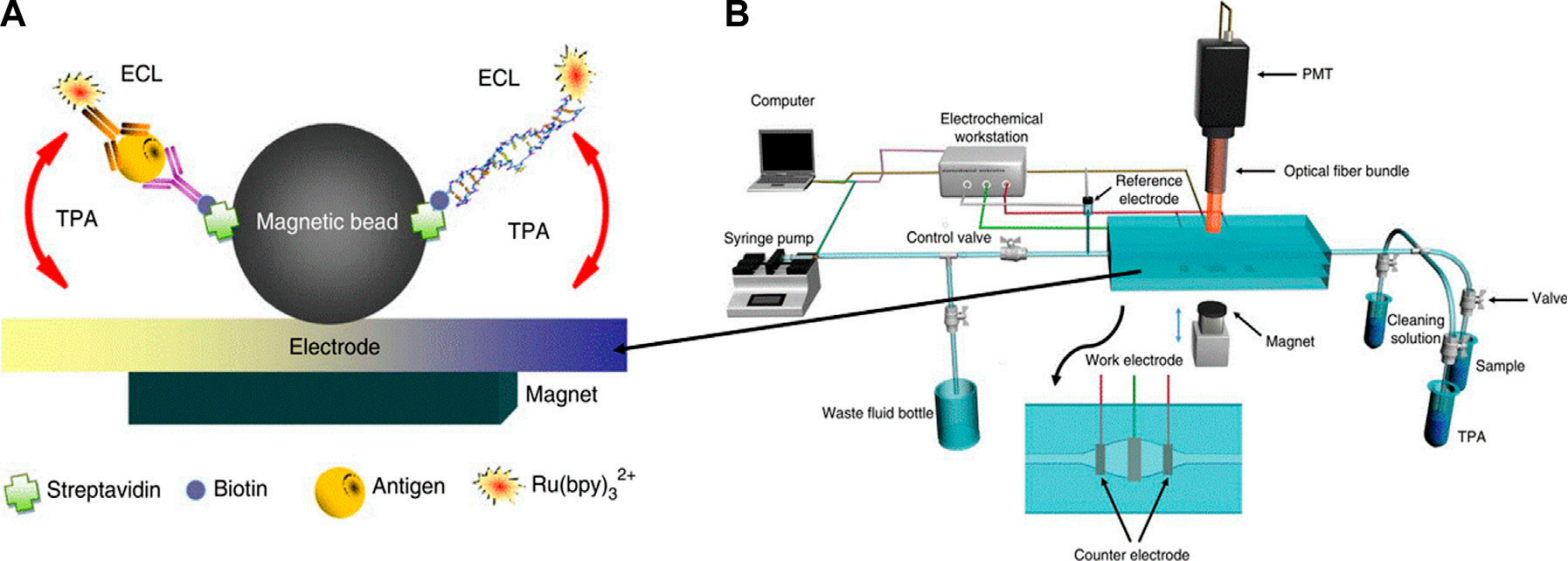
**(A)** Schematic illustration of magnetic based (MB) ECL for hepatitis B antigen model and nucleic acid target ,whereas antibody of nucleic acids are labeled with biotin and Ru(bpy), **(B)** MB based ECL measurement and detection system (Zhou et al. 2014).

In this technique, chemical luminescence and electrochemical processes are combined and named as electro-chemiluminescence (ECL), which results in the emission of light. This photo emission occurs owing to the excitation and de-excitation of electrons between the ground and excited states stimulated by the electrochemical reaction in solution. Light is emitted because of the transfer of exergonic electrons at the electrode surface. This phenomenon is also known as electrogenerated chemical luminescence. The wavelength of the light emitted from the excited and relaxed states corresponds to the energy gap of the molecules. ssDNA is commonly used as a bioreceptor and printed screen, and pencil graphite glassy carbon, and gold are used as the working electrodes in the assembly of DNA sensors. Figure 9 shows the simple strategy and mechanism of detection. This design comprises a target DNA fragment that hybridizes with MBs, where streptadivine and biotin are used as linkers, and the ruthenium probe is conjugated at the other end (Figure 9A). This magnetic bead-assisted ECL reaction occurs in the presence of tripropylamine, where the ruthenium probe Ru (bpy)_3_^2+^ amplifies the signal isothermally (Zhou et al., 2014).

Table 5 presents an overview of the various ECL-based DNA detection techniques reported in the last decade. Herein, we summarize the detection limit of 19.05 aM for biosensor based on the *in situ* generation of Cu nanoclusters as luminophores and TiO_2_ as a coreaction accelerator (Liao et al., 2018). Zhang et al. (2020e) constructed MXene (Ti_3_C_2_T_x_)-based impedimetric aptasensing nanosheets and iron phthalocyanine quantum dots for an enhanced high detection sensitivity of up to 4.3 aM as compared to the individual component-based ECL technique.

**TABLE 5:** ECL-based DNA detection techniques.

Type of affinity assay	Determination method	LOD	DLR	Ref (s)
AuNPs/Polyamidine/CuInZnS QDs	SPR/ECL/demonstrated novel SPR enhanced ECL. PDA control the separation length and enhanced ECL response, potential charge transfer and ECL quenching- P55 gene detection	0.03 nm/L	1–15 nM/L	Liu et al. (2018)
CdS nanocrystals and gold nanoparticles	ECL/we firstly enunciated the presence of synergy effect between the electron and energy transfer in an ECL system involving the CdS NCs and Au NPs for detection of the DNA binding protein	5pM	0.015–150 nM	Wang et al. (2013)
MoS_2_ nanosheets/sulfur doped boron nitrogen QDs	ECL/Demonstrated distance-dependent plasmon-enhanced ECL in detail with different length DNA chains	0.17 pmol/L	0.5 pM–1 nM	Liu et al. (2020e)
Peptide nucleic acid/core-shell Fe_3_O_4_-Au nanoparticles/	EC/miRNA sensing strategy based on the specific affinity using Fe3O4–Au–PNA probe as a new carrier in a solid-state nanopore	10 nM	2 nM–50 nM	Wang et al. (2019a)
Gold disk electrode/piranha solution	EC/Demonstrated signal amplification strategy based on copper-free click chemistry-mediated cyclic ligation of DNA	7.7 fM	25 fM–100 pM	Bi et al. (2020)
Screen printed carbon electrodes/RGO	EC/Investigated the influence exerted by the concentration of GO dispersion as a modifier for screen printed carbon electrodes on the fabrication of an EC biosensor to detect DNA hybridization	100 nM	10 nm to 10 µM	Chiticaru et al. (2019)
PDA + compounds/N, N-bis (2-(trimethylammoniumiodide) (propylene perylene-3,4,9,10-tetracarboxyldiimide (PDA+)	PEC/EC dual-mode biosensor with cationic decorated multifunctional DNA spheres *in situ* generated on electrode was proposed for sensitive and accurate detection of mRNA	0.37 fM/PEC	0.1 fM–1 nM (for PEC)	Deng et al. (2020)
		0.67 fM/EC	2 Fm–500 pM (for EC)	
Ti_3_C_2_T_x_@FePc QDs	Electrochemical/construct a novel ultrasensitive impedimetric aptasensing system	4.3 aM	0.01 fM −10 pM	Zhang et al. (2020e)
Ga2Te3-based QD/Cs+/Li+/	EC/In this study a Ga2Te3-based QD genosensor together with metal ions was developed	0.4 pg/mL	0.1–1 ng/mL	Fuku et al. (2020)
Antimonene/functionalized with an oligonucleotide/AuE	SPE/electrochemical/demonstrated that antimonene interacts non-covalently but strongly with oligonucleotides	28.3 pg/uL	0–25 ng/uL	García-Mendiola et al. (2020)
AuE/exosome/Probe DNA/hpDNA	EC/DPV- herein a sensitive hybridization chain reaction electrochemical assay was fabricated for the detection of exosomal microRNA-122	53 aM	0.1 fM to 0.1 μM	Guo et al. (2020)
Carbon Nanotube-Gold Nanoparticle Nanoclusters/	Electrochemical/biosensor combine the synergistic properties of both CNTs and AuNPs, as a promising signal amplification strategy for DNA detection	5.2 fM	0.1 pM–10 nM	Han et al. (2020)
AuE/Alumina Slurry	Electrochemical/proposed biosensor based on nest hybridization chain reaction initiated by the hybridization of two dumbbell-shaped DNA units	3 pM	5 pM–0.5 nM	Huang et al. (2020)
Poly o-cresophthalein complexone film/Glossy Carbon Electrode	EC/fabricated POCF modified electrode was and used as a sensor for the simultaneous detection of adenine and guanine as oxidation peak currents	0.02 μM	0.08 μM–200 μM	Jayadevimanoranjitham and Narayanan (2020)
Gold E/8-hydroxy-2′-deoxyguanosine	EC/Introduced triple signal amplification strategies were introduced to enhance the sensitivity of 8-OhdG	24.34 fM	100 fM–10 nM	Jia et al. (2020)
CdTe QDs Carbon ink and solid wax/electrochemical	EC cloth-based DNA sensors are developed based on Carbon ink- and solid wax	8.74 fM	20 fM to 5 nM	Jiang et al. (2020a)
SiO_2_/AuNPs) barcode/gold label silver	EC/method based on bio-barcode/gold label silver stain dual amplification is presented	0.23 fM	1 fM–10 pM	Jiang et al. (2020b)
Nanowires of polypyrrole	EC demonstrate the potential of nanostructured polypyrrole formed by template free as platform for amperometric detection of DNA	0.36 aM	1 aM–100 fM	Khoder and Korri-Youssoufi (2020)
Cu–Ni@N, B rGO (GO)	EC/Demonstrated electrocatalytic performance of Cu–Ni@N,B rGO toward guanine (G) and adenine (A) oxidation	0.118 μM	1–160 μM	Lei et al. (2020)
Carboxylate-Zr^4+^-phosphate/AgNPs	EC/proposed detection based on electrochemically mediated atom transfer radical polymerization and surface-initiated reversible addition-fragmentation chain transfer polymerization cascade polymerization and AgNPs deposition	0.487 aM	1 aM–10 pM	Li et al. (2020c)
Cu NCs/TiO_2_/ECL	ECL/biosensor based on *in situ* generation of Cu NCs as luminophore and TiO_2_ as coreaction accelerator	19.05 aM	100 aM–100 pM	Wang et al. (2019a)
PFO polymer dots/ECL	ECL-based biosensor demonstrate OH -dependent ECL emission characteristic that detect mRNA	12.2 aM	50 aM–1.0 nM	Liu et al. (2020a)
Ferrocene/Au electrodes/Al_2_O_3_/electrochemical/DPV	ECL/sensing strategy utilizing cooperative proximity hybridization based on a G-quadruplex probe labeled with the thiol	2.82 × 10^−15^M	1 nM–1 fM	Liu et al. (2020d)
PolyA-ODNs/Rolling Motor/GTD/Electrochemical	3D-ECL/Demonstrated DNA probe bridge act as catalytic center during sensing	0.17 nM	0.5 nM–1.5 μM	(Z.Liu et al., 2020)
Peptide nucleic acid/poly-L-lysine/ethyl glycol/dibenzocyclooctyne/Quartz Crystal Microbalance	ECL/presented the potential gain in sensitivity by the application of azido-PNA probes clicked to a PLL-OEG-DBCO adhesion layer adsorbed on Si-micropillar substrates at various pitches	(9.0 ± 0.2) pmol/cm^2^		Movilli et al. (2020)
		10.6 factor		
GO-wrapped Au nanostars/glossy Carbon electrodes	EC-DPV/sensing platform inspired by a functional “green” electrochemical reduction pathway	1 × 10^−20^ M	1 × 10^−20^ M-1x10^−12^ M	Rahman et al. (2020)
Trivalent Mg^2+^ dependent DNAzymes	Electrochemical Detection based on formation of trivalent DNAzyme junctions through a target-initiated catalytic hairpin assembly approach	0.46 fM	1 fM to 1 nM	Ren et al. (2020)
Capture Probe mercaptohexanol/hexanedithio monolayer	Electrochemical/designed genosensor based on mixed-self-assembled monolayers as DNA inmobilization system	10 nM	5·10–10 to 5·10–8 M	Sánchez-Paniagua et al. (2020)
CdTe QDs/Methylene blue-labeled aptamer	EC/PEC/proposed a ratiometric aptasensing strategy based on the dual-detection model with a PEC “signal-on” and an EC “signal-off”	10 nM	0.03–100 μM	Shen Z et al. (2020)
Nitrogen-doped reduced GO/glassy carbon electrode	Electrochemical/based on N-RGO/GCE sensor demonstrated electrochemical response toward the oxidation of guanineguanine	1.38 × 10^−7^ M	4.14 × 10−7–3.71 × 10−4M	Song et al. (2020a)
pencil graphite electrodes	EC/presented enzyme-linked DNA hybridization assay using PeGE to detect target DNA sequences in DNA fragments amplified by PCR	40 fM	0–50 ng/uL	Špaček et al. (2020)
Ti working electrode with electrodeposited Au nanostructures	EC (DPV) reported on-chip biosensor of DNA hybridization using Au NCs working electrodes	0.97 fM	10 fM–1 µM	Tripathy et al. (2019)
Nanoparticle gold ink on planar substrates Cyclic Olefin Copolymer (COC)	Electrochemical/Designed inkjet-printing of Au NPs at planar substrates of cyclic olefin copolymer as hybridization signal probe	60-fold higher		Trotter (2020)
Copper-based metal–organic framework/graphene nanocomposite/GCE	Electrochemical/DPV/Designed Cu-MOF/ERGO/GCE electrode for the detection of guanine and adenine in real samples	0.02–10 µM 0.005–20 µM	20–100 µM (for guanine)	Wang et al. (2020)
			40–200 µM (for adenine)	
Beacons ferrocene (Fc)-A1/methylene blue -A2	Electrochemical/Designed DNA circle capture probe with multiple target recognition domains was anchored at the top of tetrahedron DNA nanostructure	miRNA-21–18.9 aM and miRNA-155–39.6 aM	0.1 fM–10 nM	Xu et al. (2020b)
Ferrocene/graphene	Electrochemical/developed a novel tetraferrocene used as homogeneous sensor probe label that provide a greater signaling potential	8.2 fM	20 fM–2 nM	Yin et al. (2020)
Hairpin DNAN	Electrochemical/developed a novel sensor *via* target-induced Cas12a cleaving interfacial hpDNA	30 pM	50 Pm–100 nM	Zhang Y et al. (2020)
Naphthyl phosphate. Dithiothreitol, dimethylamino propyl carbodiimide	Electrochemical a new electrochemical mmune-DNA sensing platform for DNA Mtase activity assay and inhibitor screening by catalysis-based signal amplification	0.039 Um/L	0.05–10 U m/L	Yin et al. (2020)
Nanonets of GO/Fe3O4/β-CD/PAMAM-avidin-ALP	Electrochemical DNA detection by using host-guest nanonets of GO/Fe3O4/β-CD NCs as Ab platform and PAMAM-avidin-ALP as signal amplification due to electron transfer	3.2 pM	0.01–50 nM	Zhou et al. (2019)
Gold coated magnetic nanospheres	Electrochemical/Develop 3D magnetic DNA nanospheres were synthesized and immobilized on a gold stir-bar as encoded probes for miRNA capture and signal amplification	1.5 fM	5 fM–2 nM	Shen X et al. (2020)
Rethenium tris-(bipyridine)/ECL	ECL/developed sensor for 8-oxodGuo activity assay using spermine conjugated ruthenium tris-(bipyridine) derivative (spermine-Ru)	1 lesion in 500 DNA bases	0–4 U/uL	Shen Z et al. (2020)
Gold Nanocluster-H_2_O_2_ system	ECL/based sensor was fabricated for the quantification of 5 mC, TET1 protein and β-GT activities, as well as inhibitor screening, based on the interaction of chemically excited AuNCs with H_2_O_2_	3.46 pM	0.01 Nm–50 nM	Jiang et al. (2018)

##### 2.2.2.2 Microfluidic/PCR chip-based DNA sensors (MFCs)

**FIGURE 10:**
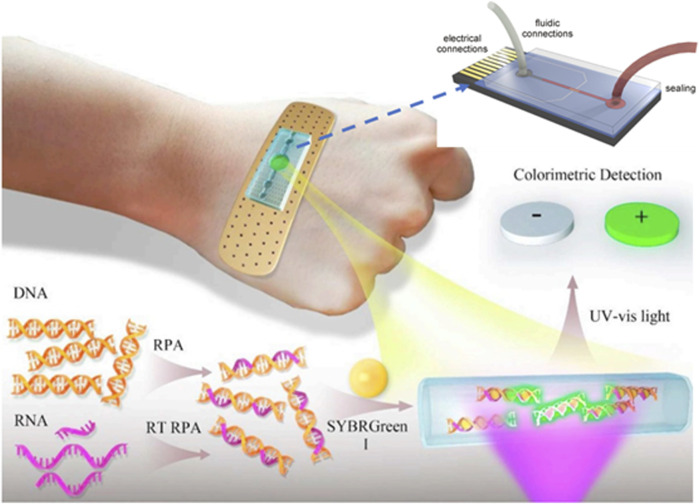
Schematic of the wearable microfluidic sensor for nucleic acids (Temiz et al., 2015; Yang et al., 2019).

Lab-on-a-chip (LOC) is a mini-integrated chip with small sensors in an array with an area of a few square centimeters. They are based on the micro-electrical-mechanical-based technology (MEMS) in the form of an integrated chip. These sensors are composed of a network of microfluidic channels in which the analyte can be manipulated at the microscale level. The compact design of microfluidic chips results in rapid heating and mixing; hence, providing ultrahigh sensitivity and portability for direct analysis of a crime scene (Temiz et al., 2015; Bruijns et al., 2016). This will also decrease the amount of analyte as well as reduces cross-contamination in the sealed environment. These systems are designed for single use, which will benefit from the chain of custody and contamination risk. A bandage-like flexible sensor that amplifies the DNA detection signal using microfluidic technology is shown in Figure 10. These sensors can be powered by body heat; thus, they are highly sensitive and portable for on-site detection (Bruijns et al., 2016). They are widely used for DNA amplification process and accelerate PCR process. They are small in dimension and instant detection and cost-effective instrument.

An overview of the various microfluidic chip-based DNA detection techniques reported earlier is listed in Table 6. We can observe that biosensors based on microfluidic chips are designed as power-free chips. They follow the amplification of fluorescence signals after hybridization with laminar flow-assisted dendritic cells and have a detection limit of 0.045–0.45 pM (Kim et al., 2019). Microfluidic-based nucleic acid amplification tests as noise resistant quantitative PCR are used for rapid detection of ultralow-abundance DNA in real biofluids. They have the highest diagnostic limits of 0.05 aM to be reported for a microfluidic technique (Ye and De, 2017).

**TABLE 6:** Chip-based microfluidic DNA sensor detection techniques.

Type of affinity assay	Determination method	LOD	DLR	Ref (s)
Glass substrate/Negative photoresist/Polydimethylsiloxane (PDMS)	Microfluidics/Fabrication of microfluidics structure-based polydimethylsiloxane biopolymer on a glass substrate with SU-8 photoresist for label-free detection DNA	260–280 nm absorbance	0.000005 A difference at 1.0 V after hybridization	Ayoib et al. (2020)
CMOS sensor for imaging	Microfluidic/Presented centrifugation assisted precipitation method for single-step DNA quantification	10Mng/uL	10–100Mng/uL	Banerjee et al. (2019)
Agarose gel electrophoresis/PCR	Microfluidic/PCR/designed 3D-printed microfluidic chip was and fabricated for droplet-based PCR detection of mRNA	normalized intensity-0.82–0.92		Jiao et al. (2019)
Ion concentration polarization	Microfluidic Electrokinetic/Sensitive detection of nucleic acids *via* hybridization on a microarray	1 nM	0.1–100 nM	Kim et al. (2020)
PDMS	Microfluidic Chip/Designed Power-free Chip in which amplification of Fluorescence signals after hybridization by laminar flow-assisted dendritic	0.045–0.45 pM	1 fM–10 pM	Kim et al. (2019)
PDMS/Poly (methyl methacrylate) (PMMA)	Flexible microfluidic chip/developed a wearable microfluidic device combined with RPA for simple and rapid amplification of DNA using human body heat	100 copies/mL in 24 min	10–10^5^ copies/mL	Kong et al. (2019)
PDMS/Polyethylene (PE)	Microfluidic PCR/propose a bubble-free microfluidic PCR device *via* controlled fluid transfer	47 copies/single PCR in <3 min	250–1,000 nM	Lee et al. (2019)
Laser diode/PMMA	Microfluidic/Proposed Disposal disc based on a double rotation axes centrifugal microfluidic platform	100 copies/mL	0–500 μL	Li et al. (2019a)
PDMS	Microfluidic PCR/proposed hierarchical selective electro kinetic concentration PCR as noise resistant quantitative PCR for rapid detection of ultralow-abundance DNA in real biofluids	0.05 aM	0–150 mL	Ye and De (2017)
Cyclic olefin copolymer (COC)	Microfluidic/based combination of online dynamic magnetic extraction procedure with droplet-based digital PCR	15.45 ng/mL	10–100 ng/mL	Perez-Toralla et al. (2019)
COC	Microfluidic/RNA was hybridized with capture probes on the reaction chamber surface and identification was achieved by detection of fluorescence tags	757.86 fM	10-1-10^−5^ pM	Prada et al. (2019)
Polycarbonate/lithium niobate	Microfluidic chip/based on non-equilibrium ionic currents and detects the presence of negative charge on target molecules	1 pM	0.001–1 nM	Ramshani et al. (2019)
Graphene Oxide (Gox)/Fluorimetric	Fluorimetric-Paired-Emitter-Detector-Diode based on GO high affinity toward single-stranded DNA and its ability to quench the fluorescence	0.625 uM	0.625–2.5 uM	Ziółkowski et al. (2020)
Au/Fe_3_O_4_ NPs/Polydopamine based microbeads	Flow cytometry/Synthesizes Au NPs decorated magnetic MBs exhibit fluorescence signal of each MBs could be collected individually, realizing single MBs-based DNA imaging	0.1 nM	0.2–20 nM	Li et al. (2020a)

##### 2.2.2.3 FET-based DNA sensors

**FIGURE 11:**
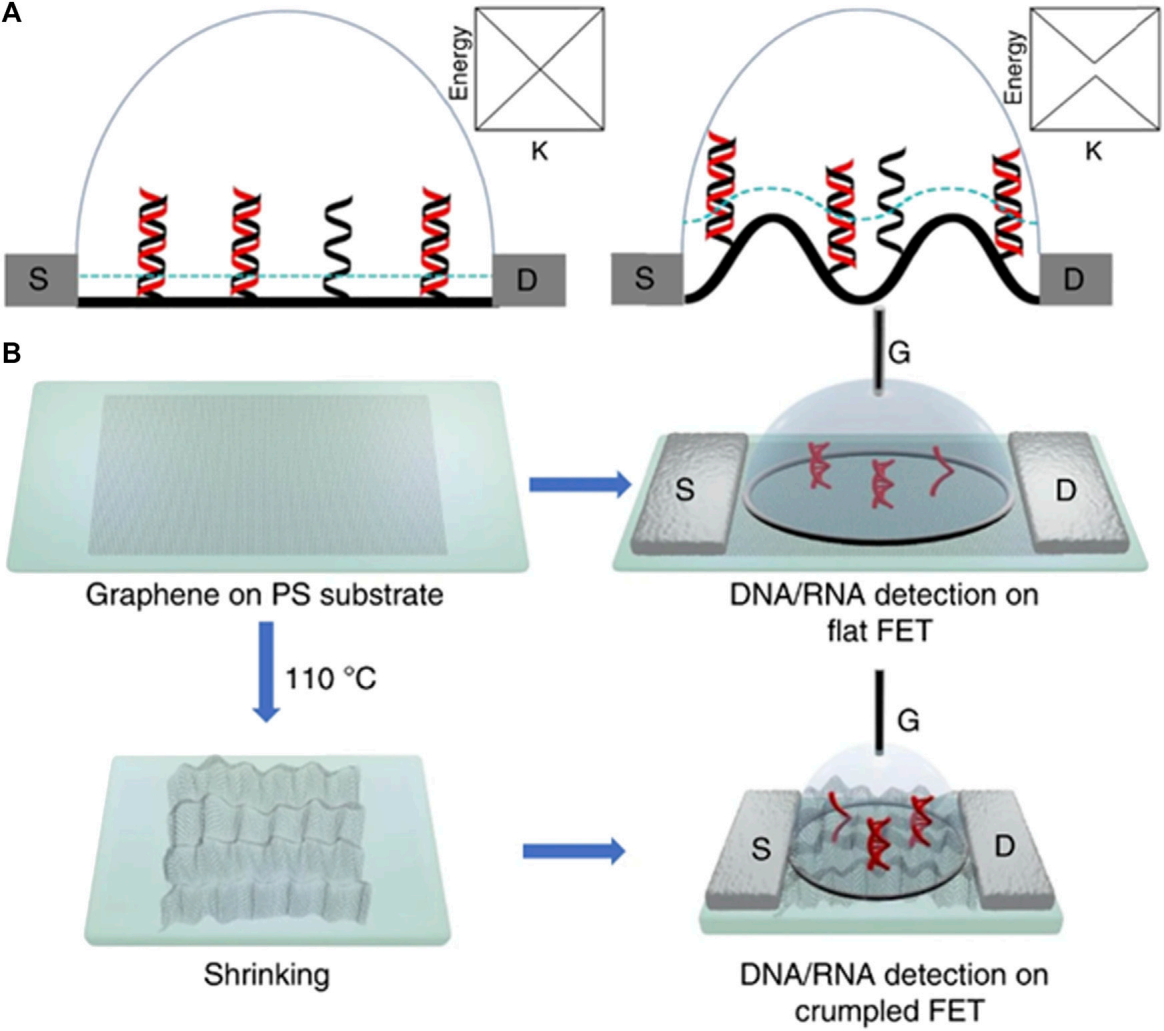
**(A)** Scheme of flat and crumpled graphene FET biosensor using single and double DNA strands over graphene surface. **(B)** Experimental route for fabrication and flow proces.

FET biosensors are highly sensitive detectors based on 2D materials. They have high carrier mobility owing to their nanoscale dimensions and high volume-to-charge ratio. MXene-based biosensors are a combination of metal carbides and nitrides that have attracted attention because of their unique characteristics (Babar et al., 2022; Guo et al., 2011). They are used for analyte detection and biosensing applications (Yadav et al., 2021). A combination of MXene-graphene-based FET has been used to detect influenza and 2019-Ncov (Li et al., 2021). A multiscale computer simulation method and experimental approach were employed to investigate the characteristics of flat and crumpled graphene-based biosensors by Hwang et al. (2020) ,who found that the detection limits of buffer and human samples are 600 aM and 20 aM, respectively. Furthermore, the atomic-scale simulation results revealed that the deformation mechanism resulted in electrical hotspots in the channel. This technique can be used to develop reliable fast-track biosensors for medical applications. Figure 11 displays a cross-sectional representation of flat and crumpled graphene FET sensors over a Debye length, represented by the blue curve (Figure 11A), whereas Figure 11B shows the fabrication route. Debye screening is weaker in crumpled graphene, which makes it more sensitive for detecting DNA (Hwang et al., 2020). The insets show the distribution of energy over the K-space.

**FIGURE 12:**
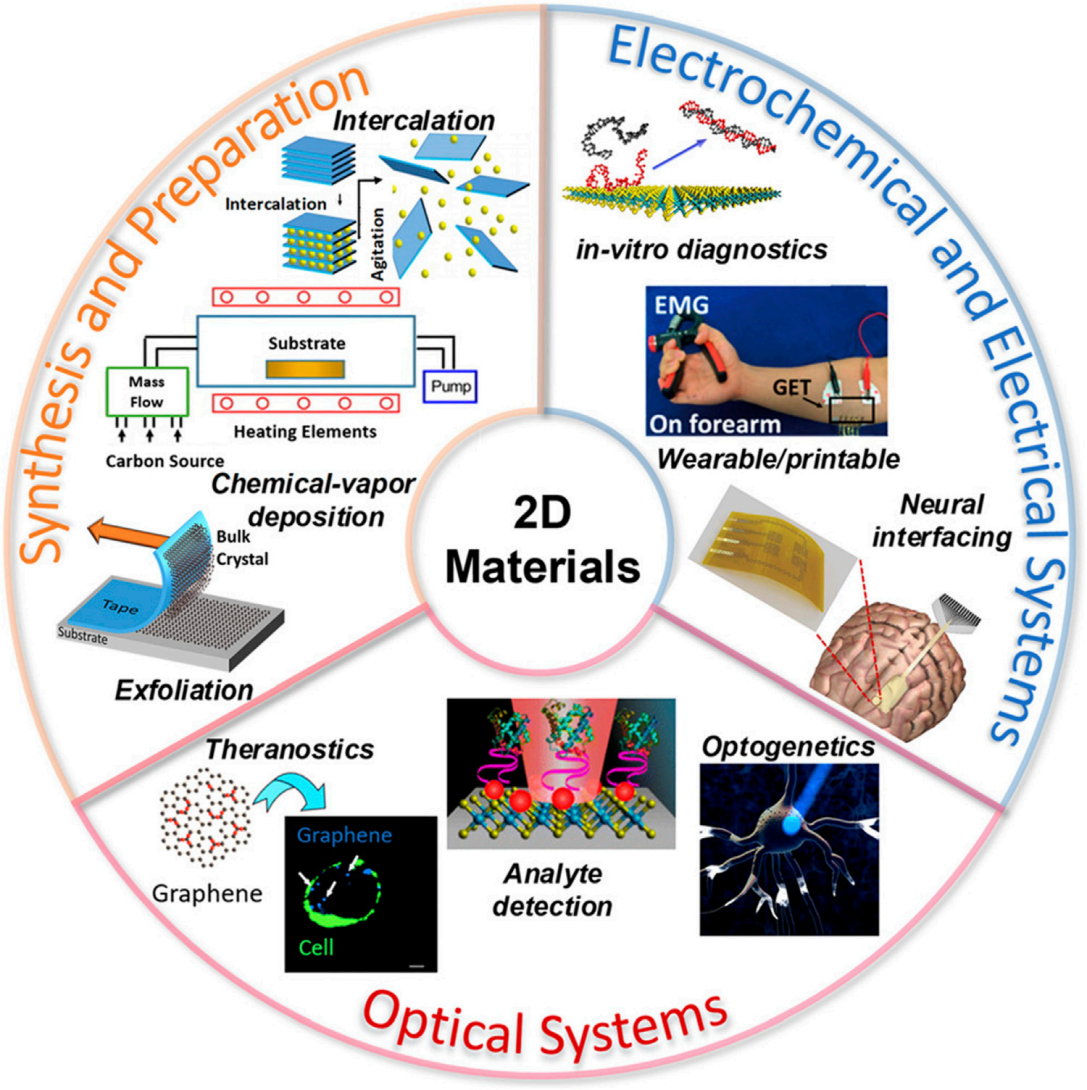
Preparation and synthesis of LD materials for optical and electrochemical applications (Bolotsky et al., 2019).

A computational study on single-layer MXene that has potential applications as a DNA detection material with high sensitivity and effectiveness was conducted (Yadav et al., 2021). Computer simulation methods have frequently been used to understand the mechanisms and interactions of materials at the atomic scale (Mustafa et al., 2017; Azeem et al., 2018a; Azeem et al., 2018b; Azeem et al., 2019b; Azeem et al., 2019a; Azeem et al., 2020; Azeem et al., 2021; Mustafa Azeem et al., 2018; Mustafa Azeem et al., 2019). An overview of the various FET-based DNA detection techniques reported earlier is listed in Table 7. It is important to point here that LD material has displayed clear advantage on other materials used for designing sensors for health application because of their tunable band structure and ultrathin nature. They also display an improved detection sensitivity. LD materials have been emerged as promising candidate for health industry. Figure 12 summarize LD materials and their application in sensing applications. The division is based on the synthesis, preparation methods, electrochemical and optical properties. The fabrication process of these materials involves process of exfoliation that depends on the chemical environment. Moreover, intercalation constitute chemical vapor deposition (CVD) and electrochemical exfoliation. The material based on LD sensors are used in theranostics image guide application and diagnostics (Bolotsky et al., 2019).

**TABLE 7:** FET-based DNA detectors.

Type of affinity assay	Determination method	LOD	DLR	Ref
Silicon nitride on SiO_2_ Substrate	Ion-Sensitive FET/CMOS chemical sensing array operating in current mode for real-time ion imaging and detection of DNA amplification by Isothermal Loop-Mediated	1.03 μA/pH		Miscourides et al. (2018)
Graphene layer on SiO_2_/Si Substrate	FET/based on two-dimensional channel of a single graphene layer that can discriminate a single nucleotide polymorphism	25 aM	1 Am–100 fM	Campos et al. (2019)
ISFET chemical frame/Bio Chip	Ion-Sensitive FET/DNA amplification generates protons H+ proportional to the number of DNA copies by isothermal amplification	16.7 mpH	1–12 pH	Abdulwahab et al. (2018)
liquid exfoliated graphene	FET/Immunorecognition based on the glutaraldehyde modified liquid exfoliated graphene FET measured by current between the electrodes of drain and source	Sensitivity <3	1–10^6^ pM	Zhang et al. (2019b)
3D Graphene	FET/by introducing target miRNA affects the electrical potential of 3D-G and detection of miRNA	100 pM	100 pM–100 nM	Song et al. (2020b)
Graphene on SiO_2_/Si Substrate	FET/Trend of Dirac point shift by adsorption of single-stranded DNA studied		Dirac Voltage Shift 10 V	Yi et al. (2019)
Graphene on SiO_2_ Substrate	FET/Back-gated G-FET-based on engineered hairpin probe DNA with improved sensitivity up to fM level	<10 fM	1,000 nM–10 fM	Gao et al. (2018)
Graphene/Magnetic bead	G-FET/that uses clustered regularly interspaced short palindromic repeats technology to enable the digital detection of a target sequence	1.7 fM		Hajian et al. (2019)
Metal/SiO2/Ion-sensitive layer	MOSFET/detected DNA by changing dielectric constant and then obtaining its threshold voltage	0.65 V		Goel et al. (2018)
AlGaN/GaN on Si Substrate	Transistor/proposed an electrical double layer gated high electron mobility transistor as DNA sensor	1 fM	1 pM–10 fM	Chen et al. (2018)
Graphene/DNA tweezers probe	FET/Single nucleotide polymorphism sensitivity achieved by observing changes in Dirac point shift and resistance change	100 pM	10 uM to 100 nM	Hwang et al. (2018)
Single-walled carbon nanotubes (CNT) on SiO_2_	FET/Developed floating electrode-based DNA sensor with controllable responses based on Langmuir theory	100 fM	1 nM–10 uM	Kim et al. (2012)
MoS_2_/Go/FET on Cu Substrate	FET/DNA detection by charge transfer through MoS2 between graphene and DNA	10 aM	10 aM–100 p.m	Chen et al. (2020)
AuNPs/single-walled carbon nanotube (SWCNT) on SiO_2_	FET/DNA detection depend on the percolation paths of SWNTs in conduction channels	100 fM	0–1 nM	Dong et al. (2008)
CNT and graphene/PCB/PAN Probe	FET/reported array of Ion-Sensitive Field-Effect Transistors for detection of nucleic acid molecules	1 nM	1 Nm–1 μM	Ganguli et al. (2018)
Graphene layer with Pyrenebutanoic acid succinimidyl ester	FET/The negatively charged effect of DNA molecule used for FET/graphene-based DNA sensor. Non-covalent bonded DNA caused a “left” shift of the Dirac point	3 nM	1–32 nM	Guo et al. (2011)
Polycrystalline Si Nanowire	FET/enhanced FET sensitivity through using chimeric DNAs with methylated neutral nucleotides as probes	0.1 fM	0.1–10 fM	Hu et al. (2018)
Crumpled/flat graphene	FET Computational simulations reveal that deformed graphene could exhibit a change in band-gap, allowing an exponential change in the source-drain current from small numbers of charges	(600 zM) [crumpled 2 pM(flat)]	10^−3^ M–10^−20^ M	Hwang et al. (2020)
CNT devices embedded in polymer substrates substrate	FET/CNT based flexible circuits for DNA sensors using Raman spectroscopy	160 nM		Kang et al. (2008)
Liquid coplaner Graphene FET	FET/Designed Liquid coplanar-gate graphene FET to detect and distinguish between single-stranded and double-stranded DNA molecules	1 nM	0–10 nM	Kim et al. (2018)
Graphene, PS Brush, SiO_2_, Si	FET/Used interfacial polymer brush layer, which is inserted between graphene and SiO2 to enhance the electrical properties of the sensor	12-mer ssDNA10	pM/μL	Ku et al. (2018)
Au electrodes/polymer substrate/PDMS/PMMA	MOSFET/developed a miRNA sensor using an electrical double layer gated FET biosensor with enhanced sensitivity and stability	100 fM	100–1,000 fM	Kuo et al. (2019)
CNT/aryldiazonium salts/Si Substrate	Designed single-point-functionalized CNTFETs have been used to sense conformational changes and binding events in nucleic acid structures from intrinsic molecular charge (Sp^3^ defects)	20-mer target DNA	100 nM	Lee et al. (2018)
Graphene/Au Gate/Glass substrate/Electrolyte	FET/The mechanism of this novel DNA sensor is that the gate potential drop is induced by DNA immobilization and hybridization on the Au gate electrode	1 fM	1 fM–5 μM	Li et al. (2019b)
Graphene/MoS_2_ heterostructures/on a sapphire substrate/Laser	Photoluminescence PL/characteristics of the grown graphene/MoS_2_ film are used for label-free and selective detection of DNA hybridization	1 aM	0.1∼1 fM	Loan et al. (2014)
MoS2/Si/SiO_2_ substrate/Ti and Au Electrode	FET/Developed label-free and direct hybridization assay using MoS2-FET biosensor for ultrasensitive detection of miRNA	0.03 fM	0.1 fM–10 nM	Majd et al. (2018)
MoS_2_/Phosphorodiamidate morpholino oligos (PMO)	FET/Developed PMO-modified MoS_2_ FET biosensor for detecting DNA based on PMO-DNA hybridization with high sensitivity and specificity	6 fM	10 fM–1 nM	Mei et al. (2018)
Graphene embedded nanochannel device/Theoretical DFT-NEGFT	Developed Graphene embedded nanochannel device that effectively controls the motion of nucleobases *via* p–p interaction and deciphers the ultrasensitivity of individual bases, one by one, in real time		Fano resonance-driven conductance of individual bases	Min et al. (2011)
Si3N4/Al on Si/SiO2Ag/AgCl References electrode	CMOS FET/based on ion-sensitive field-effect transistor array using in-pixel quantization and compensation of sensor non-idealities	3.2 μs/pH	12.8 ns–33.1 ns	Moser et al. (2018)
Al_2_O_3_ film/aluminum Floating Gate electrodes/polyethylene terephthalate substrates	FET/presented electronic transduction of DNA hybridization by coupling OCMFETs and hairpin shaped probes	100pM	10 nM–10 pM	Napoli et al. (2018)
Si_3_N_4_/doped Si substrate/Si nanonets (SiNN)/nanowires	Si nanonet FET/reported field-effect silicon nanonet transistors for DNA sensing	30/30 tested devices		Nguyen et al. (2019)
Graphene/SiO2/Si substrate/Au/Cr electrode	FET/developed a COVID-19 FET sensor in which the SARS-CoV-2 spike antibody is conjugated to a graphene sheet, which is used as the sensing area	2.42 × 10^2^ copies/mL	50–100 copies	Seo et al. (2020)
Fluorescence	FET/presented DNA sensor based on graphene and magnetic nanoparticles	1 pM	1 pM–10 nM	Sun et al. (2019a)
Carbon nanotube/Pd electrodes/SiO_2_ substrate	FET/a suspended CNT based FET was fabricated by utilizing the surface tension of liquid silver to suspend a CNT between two Pd electrodes for the detection of DNA hybridization	10 aM	1 pM–10 aM	Sun et al. (2019c)
Monolayer graphene/Ag/AgCl electrode/ITO glass substrate	G-FET/Using the graphene as the electric channel, fabricated G-FET sensor that can be used for detection of RNA	0.1 fM	0.1 fM to 1 pM	Tian, (2018)
Liquid exfoliated graphene/solution-gated FET/silver paste	FET/fabricated bioLEG-SgFETs and demonstrate their bio-application in single-strand DNA detection	10 fM	10-3-105 pM	Wang and Jia (2018)
Silicon wafer FET	FET/fabricated side-gated silicon nanowire FET was using complementary metal oxide semiconductor technology	0.83 fM	10 fM–10 nM	Wu et al. (2018)

## 3 Challenges and commercial applications

Sensors play an important role in our daily life specifically in the healthcare sector as DNA detection and its diagnostic involve curing diseases. The current trend and developing sensors are now hot topics. They have increasing demand after the nCov-19. A big challenge involving the development of DNA detection sensors requires components with on-site and rapid detection and detection limit. Before commercialization, it is very important to handle the stability and reproducibility of the samples. Another major challenge concerns the storage as handling the short lifetime of the samples and re-usage. The conventional detection methods are based on PCR techniques which are very expensive and time-consuming. It is one of the big challenges that involve the detection of ultra-low concentrations of any analyte. Another challenge involves the response time for detection. Developing accurate and economical DNA sensors is a present concern. Presently, DNA detection with nanopore technology has resolved this issue and these materials have already displayed unique characteristics. Compared to conventional methods 2D materials-based FET sensors involve the electric field to create a charge to interact with DNA and change in current results in the form of a signal to detect.

## 4 Conclusion

Infectious diseases have posed a challenge in the past few years. Biosensing and biotechnology are emerging fields. In particular, DNA biosensors have potential research applications because of their chemical properties as well as reliable and fast detection.

We have reviewed different types of biosensors and listed the fabrication techniques and materials used to develop them. Conventional methods for developing *in vitro* diagnostics are time-consuming and require multiple trials and centralized technologies. There are diverse techniques and strategies for developing sensors, including, but not limited to, collecting samples, and implementing integrated diagnostics for biological applications. LD materials are considered as alternate to traditional materials due to their nanosized thickness and compatible nature. Currently, understanding nature of these materials in biological environment is major challenge. Critical challenges exist in transforming the optimal clinical treatment of infectious diseases from trial to translational research.
